# Evolution of genes involved in the unusual genitals of the bear macaque, *Macaca arctoides*

**DOI:** 10.1002/ece3.8897

**Published:** 2022-05-24

**Authors:** Laurie S. Stevison, Nick P. Bailey, Zachary A. Szpiech, Taylor E. Novak, Don J. Melnick, Ben J. Evans, Jeffrey D. Wall

**Affiliations:** ^1^ 1383 Department of Biological Sciences Auburn University Auburn Alabama USA; ^2^ Department of Biology Pennsylvania State University University Park Pennsylvania USA; ^3^ Institute for Computational and Data Sciences Pennsylvania State University University Park Pennsylvania USA; ^4^ 5798 Department of Ecology, Evolution, and Environmental Biology Columbia University New York New York USA; ^5^ 3710 Biology Department McMaster University Hamilton Ontario Canada; ^6^ Institute for Human Genetics University of California, San Francisco San Francisco California USA

**Keywords:** baculum, hybridization, reproductive isolation, speciation

## Abstract

Genital divergence is thought to contribute to reproductive barriers by establishing a “lock‐and‐key" mechanism for reproductive compatibility. One such example, *Macaca arctoides*, the bear macaque, has compensatory changes in both male and female genital morphology as compared to close relatives. *M*. *arctoides* also has a complex evolutionary history, having extensive introgression between the *fascicularis* and *sinica* macaque species groups. Here, phylogenetic relationships were analyzed via whole‐genome sequences from five species, including *M*. *arctoides*, and two species each from the putative parental species groups. This analysis revealed ~3x more genomic regions supported placement in the *sinica* species group as compared to the *fascicularis* species group. Additionally, introgression analysis of the *M*. *arctoides* genome revealed it is a mosaic of recent polymorphisms shared with both species groups. To examine the evolution of their unique genital morphology further, the prevalence of candidate genes involved in genital morphology was compared against genome‐wide outliers in various population genetic metrics of diversity, divergence, introgression, and selection, while accounting for background variation in recombination rate. This analysis identified 67 outlier genes, including several genes that influence baculum morphology in mice, which were of interest since the bear macaque has the longest primate baculum. The mean of four of the seven population genetic metrics was statistically different in the candidate genes as compared to the rest of the genome, suggesting that genes involved in genital morphology have increased divergence and decreased diversity beyond expectations. These results highlight specific genes that may have played a role in shaping the unique genital morphology in the bear macaque.

## Graphical abstract

The bear macaque has diverged in both male and female genital morphology from other macaques, which can contribute to reproductive incompatibility. Here, 67 candidate genes involved in genital morphology were also genome‐wide outliers in various population genetic metrics. Additionally, the mean in the candidate genes of several metrics was statistically different as compared to the rest of the genome, suggesting that genital morphology has diverged beyond expectations in the bear macaque.

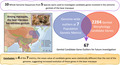

## 1 INTRODUCTION

In species with internal fertilization, genital morphology may have complex and rapid evolution. This has been demonstrated in males (Eberhard, 1993; Klaczko et al., 2015; Langerhans et al., 2016) and females (Greenway et al., 2019; Simmons & Fitzpatrick, 2019; Sloan & Simmons, 2019). There have been multiple proposed explanations for this including (1) species isolation via a “lock‐and‐key” mechanism, (2) sexual conflict, and (3) cryptic female choice (Eberhard, 1985; Sloan & Simmons, 2019). The first explanation suggests that male and female genitalia uniquely coevolve to prevent heterospecific mating. It has historically been dismissed as a general explanation for genital evolution but is acknowledged to play a role in particular cases (Eberhard, 1985, 2010). One reason for this dismissal is a presumed lack of variability in female genitalia between species, which suggests the “lock” (female genitalia) is rarely a unique match for the “key” (male genitalia) (Eberhard, 1985; Sloan & Simmons, 2019). However, studies in beetles (Sota & Kubota, 1998), flies (Kamimura & Mitsumoto, 2012), and damselflies (Barnard et al., 2017) have demonstrated reproductive incompatibility between closely related species caused by the mechanical mismatch in heterospecific crosses, suggestive of a “lock‐and‐key" mechanism (Sloan & Simmons, 2019). Sexual conflict may involve either conflict between males trying to obtain mates or conflict between males and females in levels of investment in offspring (Eberhard, 1985). Cryptic female choice happens when male reproductive output is affected by the female after copulation (Eberhard, 1985, 1996). These processes both involve competition that may also result in species‐specific genitalia. For example, a prediction of cryptic female choice is that female genitalia evolve more rapidly than male genitalia. This can result in compensatory coevolution of male genitalia that causes reproductive isolation between species (Greenway et al., 2019; Langerhans et al., 2016; Sloan & Simmons, 2019). In support of this, it has been found that male and female genitalia coevolve in internal fertilizing poecilid fishes (Greenway et al., 2019) and in dung beetles female genitalia diverge more quickly than male genitalia (Simmons & Fitzpatrick, 2019). Additionally, it has been found that females in populations of dung flies exhibiting sexual conflict have greater preference for conspecific males than females in monogamous populations (Martin & Hosken, 2003).

**FIGURE 1:**
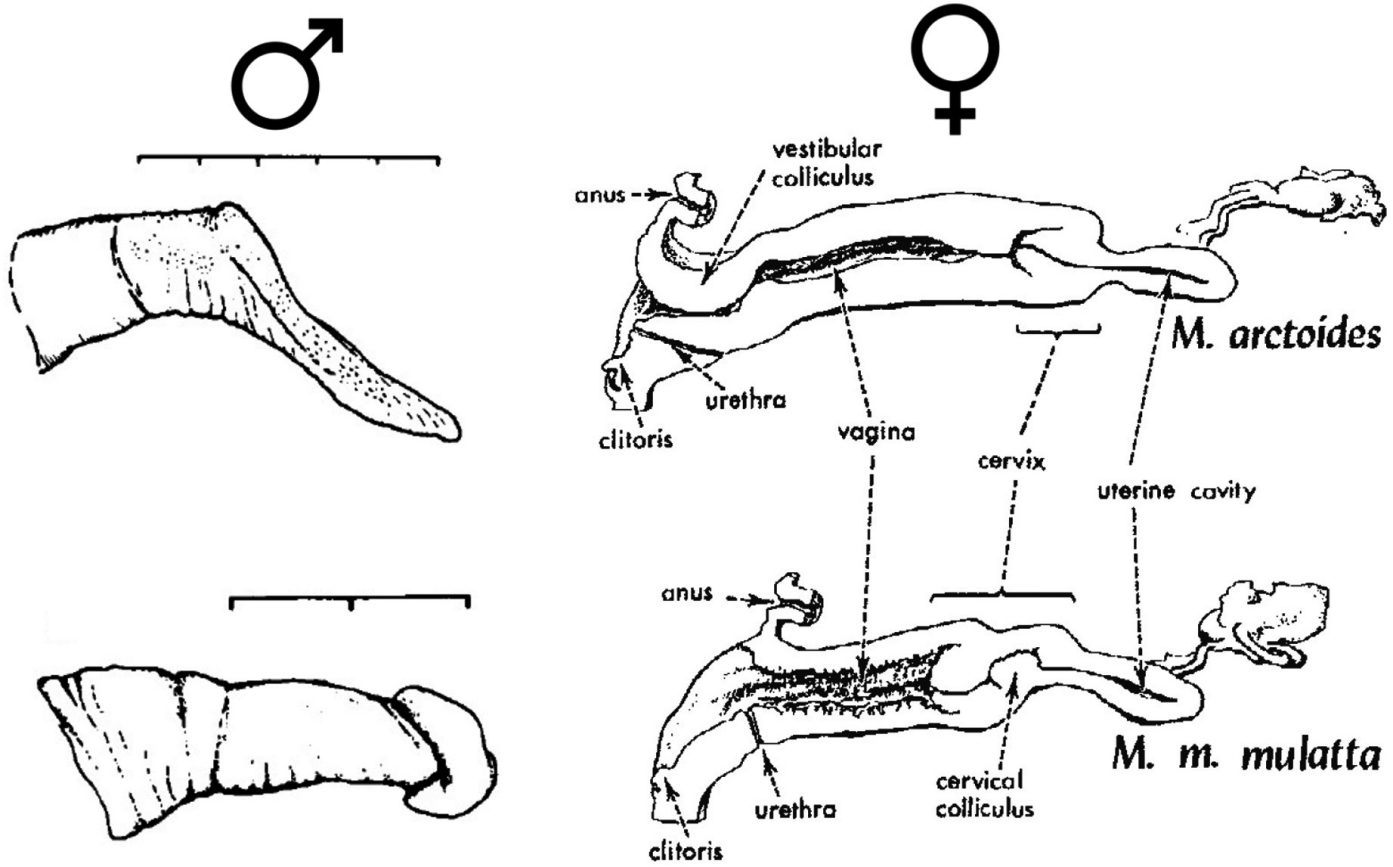
Diverged genital morphology in male and female bear macaques. *M*. *arctoides* has divergent male and female genital morphology (top), relative to *M*. *mulatta* (bottom) representing mechanical isolation between these species. *M*. *mulatta* morphology is representative of other species in macaques. Male external genitalia for each species are shown on the left (from figure 3 of Dixson, 1987) and female reproductive tracts are shown on the right (from figure 4 of Fooden, 1967). Reproduced with permission. Divergent traits for male external genitalia include size, shape, and color. Divergent traits for female reproductive tracts include depth of the uterine cavity, width of the vagina, and opening size. Both males and females differ in the urethra location. Scales on the left are in 1 cm divisions. Note: the baculum, which is an internal male genital structure, is divergent in size and shape (not pictured)

The bear macaque, *Macaca arctoides*, is unique among macaques (and primates generally) in its genital morphology, with distinctive genital characteristics in both sexes (Figure 1). In males, it has the longest baculum of any primate (4–6 cm; Fooden, 1990), even when corrected for body weight (Dixson, 1987; Fooden, 1990). In addition to its elongation, the urethral opening is located on the ventral side of the penis, altering the overall morphology relative to other macaque species (Fooden, 1990). In females, *M*. *arctoides* female genitalia appears uniquely coadapted to their male counterparts in both length and morphology (Fooden, 1967). Given these compensatory changes, it has been hypothesized that genital evolution in this species followed a “lock‐and‐key” mechanism, which is likely to be rare among primates (Dixson, 1998).

Beyond genital morphology, the mating behavior of the bear macaque is also atypical among macaques. In most other macaques, males and females mount multiply, but in contrast – and perhaps due to the altered male genital morphology – bear macaques engage in a single‐mount ejaculatory mating strategy with a prolonged sitting where the males deposit an ejaculatory plug in females (Fooden, 1990). However, most studies on macaques on mating behavior have used *M*. *arctoides* as the sole representative of the *sinica* species group (Dixson, 1987; Dixson & Anderson, 2004). When considering *Macaca radiata*, also a member of the *sinica* species group, studies have found they too favor a single mount mating style similar to *M*. *arctoides*, suggesting this is not a unique feature of this species, but instead representative of the *sinica* species group (Shively et al., 1982). Similar to *M*. *arctoides*, *M*.* radiata* also exhibits a post‐ejaculatory sit. Since both species have larger bacula than species in the *fascicularis* group, the hypothesis that increased baculum length aids in prolonged intromission is supported.

**FIGURE 2:**
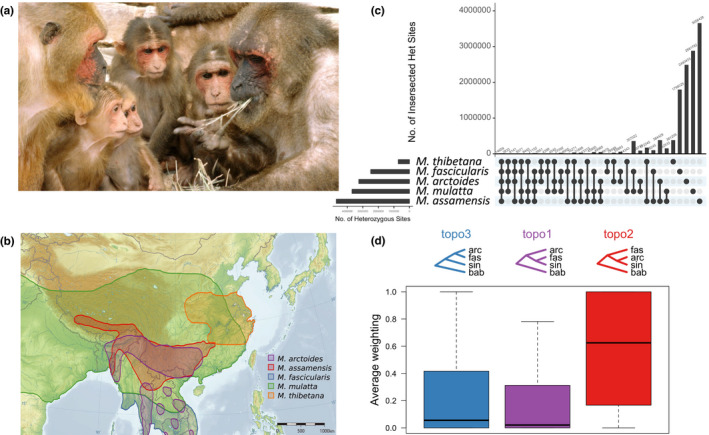
Summary of focal species, *Macaca arctoides*, its evolutionary relationships, and the data used. (a) *M*. *arctoides* has unique genital morphology and is a putative homoploid hybrid species. Image “Stumptail Monkeys” from (Waal, 2004) by Frans de Waal licensed under CC. (b) The geographic range of *M*. *arctoides* and other focal species in this study. *M*. *arctoides* has present‐day geographic overlap with members of both species groups (redrawn from Fooden, 1980). Background image “Topographic map of East Asia” by Ksiom is licensed under CC BY‐SA 3.0. This study included WGS from *M*. *arctoides* and representative members of each species group (Table 1). Baboon was used as an outgroup species. (c) The intersection of heterozygous sites between the five species. The output of vcf‐compare from vcftools (Danecek et al., 2011) was input into the UpSetR package (Conway & Gehlenborg, 2019) to plot the intersection of sites. Values in the left plot match count of heterozygous sites per sample in Table 1. (d) Topology weight distribution of three rooted topologies of species relationships in sliding windows along the genome. bab—*P*. *anubis*

Additional distinguishing characteristics of the bear macaque that are not related to genital morphology include whitish pelage in newborns, and a bald forehead and cheeks (the latter evident in Figure 2a) (Fooden, 1990). Further, the evolutionary history of this species has been noted as extremely complex with early molecular work revealing phylogenetic incongruence between mitochondrial and Y‐chromosomal genealogies for *M*. *arctoides* (Tosi et al., 2000, 2003). Mitochondrial markers place *M*. *arctoides* as sister to the *fascicularis* species group (which includes *Macaca cyclopis*, *Macaca fascicularis*, *Macaca fuscata*, and *Macaca mulatta*), whereas autosomal markers place it as sister to the *sinica* species group (which includes *Macaca assamensis*, *M*. *radiata*, *Macaca sinica*, and *Macaca thibetana*) (Fan et al., 2018; Jiang et al., 2016; Li et al., 2018; Li, Han, et al., 2009). Similarly, both morphological and Y‐chromosomal trees support placement in the *sinica* group (Fan et al., 2018; Jiang et al., 2016; Tosi et al., 2000) (see Table S1 in Fan et al., 2018 for review). This phylogenetic incongruence was hypothesized to be consistent with ancient hybridization between the ancestor of the *fascicularis* species group and a *sinica* species group member (an ancestor of either *M*. *assamensis* or *M*. *thibetana*) contributing to the putative hybrid speciation of *M*. *arctoides* (Tosi et al., 2003). In contrast to allopolyploid speciation, homoploid hybrid speciation (HHS) conserves chromosome number (Mallet, 2007; Mavárez & Linares, 2008; Schumer et al., 2014). This hypothesized hybrid origin is diagrammed in Figure S1A, with a range of possible split times indicated by the thick purple line.

A recent analysis of macaque genomes supports introgression between *M*. *arctoides* and members of the *fascicularis* and *sinica* species groups (Fan et al., 2018), both of whom it overlaps geographically (Figure 2b). These species groups are estimated to have split ~3 million years ago. Recent phylogenetic studies indicate introgression may have occurred after the split of *M*. *mulatta* and *M*. *fascicularis* (Fan et al., 2018; Roos et al., 2019), which is estimated at 1.68 mya (Fan et al., 2018; Jiang et al., 2016; Stevison & Kohn, 2009). This timing overlaps glacial periods of the Pleistocene which is associated with a reduced expanse of Southeast Asian forests into multiple refugia. Ecological isolation is a common feature of other scenarios of HHS (Gompert et al., 2006). In the case of *M*. *arctoides*, isolation is hypothesized to have contributed to the extensive interspecific hybridization, forcing the interbreeding of species that otherwise might have had alternative options for mating. Available phylogenetic evidence suggests that this interbreeding subsided ~1.5 mya (Tosi et al., 2003), with limited fossil evidence of hybridization beyond the late Pleistocene (Fooden, 1990). The evidence for historical hybridization suggests that the derived genital morphology may have been influenced by or evolved despite extensive genetic exchange with other macaque species (Tosi et al., 2000, 2003).

Here, we investigated the potential role of hybridization in shaping the unusual genital morphology of *M*. *arctoides*. We hypothesized that if the unique genital morphology contributes to reproductive isolation, then genes involved in genital morphology would show extreme population genetic signatures for *M*. *arctoides*. To test this hypothesis, we compiled a list of 2284 candidate genes associated with genital morphology in other organisms, with a specific focus on mammals. We used an existing recombination rate map for *M*. *mulatta* to identify outliers in several population genetic metrics, such as introgression, nucleotide diversity, and divergence. Phylogenomic and population genetic approaches were used on several extant macaque species (*M*. *arctoides*; the *sinica* group species *M*. *thibetana* and *M*. *assamensis*; and the *fascicularis* group species *M*. *fascicularis* and *M*. *mulatta*). We found that 67 of the candidate genes were significant genome outliers in at least one of seven population genetic metrics. Further, the mean across 4/7 population genetic metrics was significantly different in the candidate genes as compared to all other genes in the genome. Interestingly, three *dN*/*dS* outliers were associated with putative baculum morphology in mice. Overall, our results suggest that genital evolution in the bear macaque involved a large number of genes associated with various phenotypic changes in both males and females.

## 2 METHODS

### 2.1 Samples and Genome Sequencing

One female each from *M*. *arctoides* (Malaya) and *M*. *assamensis* (A20) were sequenced. The *M*. *arctoides* sample was from the Malaka Zoo in Malaysia (previously described in Tosi et al., 2000). These two samples were multiplexed and sequenced on one lane of sequencing for initial quality assessment. They were then subsequently run on one additional lane of sequencing each for a final 1.5 lanes per sample. Sequencing was done at UCSF Medical Center Sequencing Facility on an Illumina HiSeq 2000 machine. Each run was assigned as different read groups to be treated as independent in the variant calling workflow. The raw read data have been deposited in the NCBI sequence read archive and are available via the accession PRJNA622565 (see Table 1).

**TABLE 1:** Sample information

ID	species	Species group	Coverage	No. sites VQSR passed	No. sites heterozygous	NCBI accession	SAMN_ID	Sex
**Malaya**	***M. arctoides***	**arctoides**	**20**	**6,849,957**	**3,252,204**	**SRS6488501**	**14521207**	**F**
SM1	*M. arctoides*	arctoides	32	6,848,826	3,251,892	SRR2981139	04316319	F
SM2	*M. arctoides*	arctoides	19	6,833,185	3,246,931	SRR2981140	04316320	F
BGI‐CE‐4	*M. fascicularis*	fascicularis	43	4,547,767	2,486,762	SRS114988	00116341	F
CR‐5	*M. mulatta*	fascicularis	44	4,561,581	3,693,024	SRS115022	00113489	F
BGI	*M. mulatta*	fascicularis	10	4,531,187	3,678,452	SRS212016	00627571	M
BGI‐96346	*M. mulatta*	fascicularis	4	4,334,465	3,533,580	SRS114988	00113455	M
**A20**	***M. assamensis***	**sinica**	**13**	**7,567,951**	**4,680,934**	**SRS6491954**	**14521608**	**F**
XH1	*M. assamensis*	sinica	49	7,669,854	4,702,055	SRR2981114	04316321	M
Tibetan‐macaque‐NO.3	*M. thibetana*	sinica	27	4,844,002	713,449	SRR1024051	02390221	F

Note

A list of samples used in this study with a summary of genome information. Two samples were sequenced as part of this study (in bold) and the remainder were downloaded from NCBI.

### 2.2 Publicly available sequences

Raw FASTQ files were downloaded from 8 public genome samples for this project (Table 1). Genome samples were selected to be representative of the taxa relevant for *M*. *arctoides* evolution. For the *fascicularis* group, many more public sequences were available than used here, however, we selected two sequencing projects and included all samples from both projects, including those with lower coverage (<10X, see Table 1). This sample size was chosen to allow for equal representation across the various species groups. Additional sample details can be found in the corresponding publications (Fan et al., 2014, 2018; Yan et al., 2011). These were downloaded from NCBI SRA using sratoolkit version 2.8.1 with options to split data into read pairs and in the original format (ncbi/sra‐tools, 2008). Because *M*. *thibetana* was sequenced on an older platform, we used seqtk (Li, 2019) to convert from Q64 to Q33. Additionally, SRA files from multiple lanes as indicated in the FASTQ header were split for independent alignment to the reference genome facilitating the definition of separate read groups to be treated as independent in the variant calling workflow.

### 2.3 Alignment, genome analysis, and variant calling

Raw FASTQ files from all samples were aligned to the reference genome rheMac8 (NCBI Accession: PRJNA214746). The masked reference genome was downloaded from UCSC. The reference genome for rheMac8 includes 20 autosomes, a pair of sex chromosomes, a mitochondrial genome, and 284,705 unnamed scaffolds of varying length. The total length of all scaffolds is 3.2 Gb. The total length of all named chromosomes is 2.8 Gb. GATK best practices were followed to obtain high‐quality variant sites (see Supplementary Methods and Tables S1 and S2). Briefly, reads were aligned to the reference genome, duplicates were marked, indels were realigned, base quality scores were recalibrated, variants were called, and variant quality scores were recalibrated. Samples from each species were processed independently and merged into a final callset (see Data Availability). The baboon reference genome was added to the variant files via two‐way genome alignment and custom scripts that are available via GitHub. These final filtered files for each chromosome with the baboon information are freely available and were used for all subsequent analyses. A more detailed workflow is described in both the supplement as well as the GitHub page for the project, which includes command prompts and custom scripts (see Data Availability).

### 2.4 Four‐taxon test for introgression

To analyze patterns of introgression in sliding windows, a modified four‐taxon test was used (Figure S2). Rather than separately analyzing gene flow between *M*. *arctoides* and each parental species group, as has been done previously (Fan et al., 2018), we relied on the modified statistic *f_dM_* (Malinsky et al., 2015), which is suggested to perform better than other related statistics based on simulation results (Martin et al., 2015). Specifically, hybridization between the three samples of *M*. *arctoides* (P3) was tested with samples from the *sinica* species group (P1) and the *fascicularis* species group (P2), using the baboon reference genome as an outgroup (papAnu4; see Supplement for details). Our approach differed from the typical way these tests are setup where one may only be interested in shared ancestry between two pairs of taxa, (P1:P3 and P2:P3), and the P1 and P2 taxa are more closely related to each other than either are to P3 (see figure 2 in Kulathinal et al., 2009). However, this modification allowed for the quantification of shared ancestry from both putative parents together, with a single metric ranging from −1 to 1. We note that previous work had similar results when using the more traditional four‐taxon test setup for these taxa (Fan et al., 2018). To further justify this modified approach, this analysis was repeated with individual taxa from each species group (Table S3). Genome‐wide averages for both *D* and *f_dM_* were similar when a single representative of each species group was included (Table S3), suggesting that grouping the species did not qualitatively change the overall results (see Supplementary Methods).

Calculations of *f_dM_* were done using scripts available on GitHub (Martin, 2019) and using several differently sized genomic windows. *f_dM_* was computed for overlapping windows of sizes 5, 25, 50, 100, 500, and 1000 kb, with a step size of 20% of each window size. The minimum number of sites per window size was based on the distribution of sites available in the callset at each bin size. Specifically, a minimum number of sites of 10, 50, 100, 200, 1000, and 2000, respectively, per window size were used. Because it is recommended to have at least 100 sites per window (Martin, 2019), the 50 kb window size was selected and the results are presented in Figure 2 as a heatmap across the genome. Results from the additional bin sizes are shown in Figure S3.

### 2.5 Topology weighting

To analyze the weights of different phylogenetic topologies across the genome, the software Twisst was used to compare the weights of different species topologies throughout the genome (Martin & Belleghem, 2017; Martin et al., 2019). First, data used for the introgression analysis were pruned to make sure that each group had at least one member represented in the genotype data. Next, neighbor‐joining trees were constructed in sliding windows of 50 SNPs each across the genome using Phyml from scripts available on GitHub (Martin, 2019). This generated Newick formatted tree files that were used as input for Twisst. The resulting weight distributions among the three possible topologies were summed across the genome (Figure 2d).

The topology weighted outputs were further explored by collapsing adjacent intervals with the same majority topology supported. Since there were three possible topologies, the majority topology was defined as any one topology that had at least two‐thirds of the total weight values. Any intervals without a majority topology were labeled as “unresolved.” If adjacent intervals supported the same majority topology, they were collapsed. The data were subsequently split into the three major topologies. These were intersected with gene regions from the Ensembl annotations for rheMac8 downloaded from UCSC.

Bootstrap confidence intervals for topo1, topo2, topo3, and unresolved proportions were computed by resampling 2792 1 Mbp intervals along the genome (149 when considering only chromosome X) for each of 10,000 replicates and taking the 2.5% and 97.5% quantiles.

### 2.6 Divergence analysis

Based on the three possible topologies, nucleotide divergence was calculated as *D_X_*_Y_ using scripts available on GitHub (Martin, 2019). First, stringency in defining the majority topology was increased to require 100% of the weights to be on only one of the three topologies. Next, divergence was calculated between *M*. *arctoides* and members of the *fascicularis* species group (*M*. *fascicularis* and *M*. *mulatta*) for regions with topo3 ((arc,fas),sin). As well, divergence was calculated between *M*. *arctoides* and members of the *sinica* species group (*M*. *assamensis* and *M*. *thibetana*) for genomic regions with topo2 (fas,(arc,sin)). For the regions with topo1 (arc,(fas,sin)), the relative divergences were unchanged when divergence was calculated between *M*. *arctoides* and members of the *sinica* species group as compared to divergence calculated between *M*. *arctoides* and members of the *fascicularis* species group, therefore only the latter is reported.

### 2.7 Candidate gene selection

The unique genital morphology of both males and females in the bear macaque (Figure 1) led us to focus our study on candidate genes that have been identified in other studies to be associated with genital morphology. We conducted a literature search for terms associated with genital morphology such as “disorder of sex development,” “DSD,” “baubellum,” and “baculum,” the latter two terms included due to the extremely large baculum of the bear macaque, and the female equivalent structure. We filtered to remove genes that are solely associated with either fertility or adult hormonal disorders/shifts as these conditions are unlikely to affect the development of genitalia. Included in our initial list of 141 genes (Table S4) were genes associated with gonad dysgenesis (Bamshad et al., 1997; Ekici et al., 2013; Lavi et al., 2020; Witchel, 2018), female masculinization (Wang et al., 2020), genital tubercle formation (Haraguchi et al., 2000), baculum morphology (Schultz, Ingels, et al., 2016), hypogonadism (Délot et al., 2017), gonad differentiation (Witchel, 2018), gonad development (Witchel, 2018), sex determination (Délot et al., 2017), and sex differentiation (Délot et al., 2017).

In addition to our literature search, we used the Human Phenotype Ontology (HPO) (Robinson et al., 2008) database to target search terms associated with genital abnormalities, specifically targeting those that would likely be developmental. We therefore targeted the HPO category “Abnormal Reproductive System Morphology,” but removed categories that were unrelated, such as “Genital Ulcers.” A list of the HPO terms can be found in Table 2. In total, this added 1230 genes to our 141 sets of literature candidate genes. It is worth noting that 88 genes overlapped between these lists, with the remainder being unique to our literature search.

**TABLE 2:** Summary of candidate gene lists

Unique ID	Subontology	Database	# of Genes	# rheMac8 orthologs	# genes analyzed *dN*/*dS*	# genes analyzed other stats	# outliers
**n/a**	**Manually curated set**	**Literature**	**141**	**110**	**17**	**64**	**7**
HP:0000811	Abnormal external genitalia	HPO	1061	920	140	425	34
HP:0010461	Abnormality of the male genitalia	HPO	1053	907	142	424	35
HP:0010460	Abnormality of the female genitalia	HPO	512	447	72	223	16
HP:0000812	Abnormal internal genitalia	HPO	468	403	62	196	14
HP:0001827	Genital tract atresia	HPO	16	15	3	10	1
HP:0012244	Abnormal sex determination	HPO	20	17	2	8	1
HP:0003252	Anteriorly displaced genitalia	HPO	1	1	1	2	0
MP:0009198	Abnormal male genitalia morphology	MP	1145	959	130	478	23
MP:0009208	Abnormal female genitalia morphology	MP	696	587	89	304	18
MP:0002210	Abnormal sex determination	MP	782	644	90	315	18
MP:0003936	Abnormal reproductive system development	MP	99	91	12	46	1
			**Grand total**	**2284**	**313**	**1055**	**67**

Note

In addition to a manually curated list from the literature, several phenotype terms were mined from the Human Phenotype Ontology (HPO) and the Mammalian Phenotype (MP) Databases. Here, the number of genes associated with each term is listed along with the number of orthologs in rheMac8. Further, the number of genes used in our analysis where we calculated seven population genetics metrics are listed along with the final number of outliers per candidate gene set.

Additionally, because certain phenotypes are lacking in humans, such as the baculum, we did a similar search of the Mammalian Phenotype (MP) Browser (http://www.informatics.jax.org/vocab/mp_ontology) available through the Mouse Genome Database. We used largely overlapping search terms to the HPO terms, totaling a set of 1554 genes. As expected, there were several genes that overlapped across each set, but also many genes unique to each query. In sum, our final candidate gene list comprised 2284 genes from the literature, as well as the HPO and MP databases that had orthologs in the rheMac8 reference genome (Table 2). Of these 2284 genes, only a subset was used in the calculations of each population genetic metric based on the constraints of these subsequent analyses. The major limiting factor was the data for recombination rate, which was used to identify genomic outliers (see next section). The total number of genes analyzed for each statistic is reported in Table S6 and summarized in Table 2. For most statistics, 1055 candidate genes were included in the population genetic analysis for outliers.

### 2.8 Outlier analysis

With the candidate gene list, we sought to identify if any genes on our list had extreme values for diversity, differentiation, or introgression within the bear macaque. To do this, we took a gene‐centered approach. Instead of the pre‐defined windows used earlier, we used the Ensembl Gene Annotations for rheMac8 to define window coordinates. First, the smallest region containing all transcripts from the annotation file was computed. Then, 5 kb upstream and downstream was added to include potential regulatory regions for each gene. These windows were used to re‐calculate *f_dM_*, and to calculate nucleotide diversity, π. We also chose to look at two metrics for nucleotide divergence—*F_ST_* and *D_XY_*—calculated to compare *M*. *arctoides* to members of both the *fascicularis* and *sinica* species groups separately. All metrics were calculated using scripts available on GitHub (Martin, 2019). Finally, we estimated *dN*/*dS* for each gene. This methodology was previously described in Bailey and Stevison (2021). Briefly, we used the three possible gene tree topologies to estimate a branch‐specific *dN*/*dS* for the *M*. *arctoides* branch for every gene in the genome. Estimates where *dS* was 0 and *dN*/*dS* was greater than 10 were excluded as these genes had insufficient divergence to obtain valid estimates (David et al., 2020; Villanueva‐Cañas et al., 2013). Then, the topology weights for each gene window from our Twisst analysis were used to weight the *dN*/*dS* estimates for each gene across the three gene trees.

To further identify extreme values of each metric, we took into consideration that many of these statistics have been shown to be correlated with recombination rate, which can impede the detection of outliers, specifically enriching for outliers in regions of low recombination rate (Stevison & McGaugh, 2020; Wolf & Ellegren, 2017). This approach should also help to account for the potential bias of several of these statistics in regions of low diversity, which is highly correlated with the recombination rate. Therefore, we also computed an average recombination rate for each gene. For this, we download the UCSC track for recombination rate associated with rheMac8 (ftp://ftp.hgsc.bcm.edu/ucscHub/rhesusSNVs/rheMac8/all.rate.bw) (Xue et al., 2016) and converted it from bigwig to bedGraph format using UCSC utilities. Bedtools (Quinlan & Hall, 2010) was then used to calculate the average recombination rate (cM/Mb) in each gene interval.

Finally, the Cook's distance (*cooksd*) (Cook & Weisberg, 1982) was calculated from a linear regression between each statistic (*F_ST_*, *D_XY_*, *f_dM_*, *dN*/*dS*, and *π*) and recombination rate per gene (Table S6). Cook's distance provides a measure of the leverage and residual values of a particular data point, which indicates how much it influences the overall regression. Thus, it reveals how much the regression changes when that observation is removed, making it useful for identifying outliers in a regression analysis. Here, extreme values were determined based on genes that were 3*mean(*cooksd*) above or below the fitted value in the regression model, depending on the particular statistic. The resulting outlier genes were genes where the statistic being evaluated (e.g., *F_ST_* and *D* ***_XY_***) was much higher/lower than expected when considering the relationship between the statistic and recombination rate. For *f_dM_*, because it is a two‐tailed distribution, outliers from both the upper and lower end of the distribution were computed. For *dN*/*dS*, we did not find a statistically significant relationship with recombination rate, consistent with prior findings in humans (Coop & Przeworski, 2007). Therefore, we instead used a 5% cutoff for extreme values of this statistic instead of the Cook's distance approach. This percentage produced fewer genome‐wide outliers than the average of all other metrics, suggesting it as an appropriate cutoff (Table S6).

Genes identified as genome‐wide outliers were then compared against our set of *a priori* candidate genes to identify outliers that were putatively involved in genital morphology. A chi‐square test was conducted to determine if the number of outliers that were in our candidate gene set was more than expected due to random chance. A summary of the regression results and chi‐square tests can be found in Table S6, and the resulting 67 outlier genes that overlapped our candidate gene set can be found in Table S7. BCFtools (version 1.11) (Li, Handsaker, et al., 2009) view was used to extract VCFs for the 67 outlier genes and these 67 VCFs were combined and annotated using SnpEff (version 5.0) (Cingolani et al., 2012) to examine the putative impacts of variants identified within this system. The Mmul_8.0.1 reference genome and gtf annotation were used to generate a SnpEff database using protocol described on the SnpEff website (http://pcingola.github.io/SnpEff/se_buildingdb/#add‐a‐genome‐to‐the‐configuration‐file). These annotations include exons, introns, UTRs, and regions 5 kb upstream and downstream of start and end sites for genes to account for possible regulatory factors. These annotations are provided in tabular format in Table S7.

### 2.9 Permutation analyses

Permutation tests were used to identify differences in various summary statistics (*F_ST_*, *D_XY_*, *f_dM_*, *dN*/*dS*, and *π*) between candidate genital genes and all other genes. For calculations of *π*, this was a one‐tailed test where genital genes were expected to have reduced *π* in *M*. *arctoide*s compared to other genes. For calculations of *dN*/*dS* this was a one‐tailed test where genital genes were expected to have increased *dN*/*dS* in *M*. *arctoides* compared to other genes. All other comparisons (*F_ST_*, *D_XY_*, and *f_dM_*) were two‐tailed tests. In all of these tests the test statistic used was the difference between the mean summary statistic for the candidate genital genes and the mean for the same summary statistic for all other genes. These analyses were conducted using modified perl scripts from Evans et al. (2021) and the results are reported in Table S6.

In addition to this, we conducted permutation analyses on genes categorized as being involved in either male or female genital development or abnormalities according to the HPO and MP databases. Specifically, the categories used were HP:0010461, HP:0010460, MP:0009198, and MP:0009208 (see Table S5). This was done to examine the possibility of male and female genital morphology evolving at different rates (see Introduction). These analyses parallel the above in examining the same summary statistics, though all tests were two‐tailed as there was not a specific expectation for the direction of evolution.

## 3 RESULTS

Genomic analysis of five macaque species revealed a mosaic of evolutionary ancestry with respect to two major species groups. These analyses used newly sequenced whole genome samples from *M*. *arctoides* (20X coverage) and *M*. *assamensis* (13X), and 8 publicly available genomes ranging in coverage from 4 to 49X (median 32X; Table 1). Most variable positions were heterozygous in only one species (Figure 2c). For shared variants, *M*. *arctoides* shares the most variants with the *sinica* species group, *M*. *assamensis* and *M*. *thibetana*, consistent with taxonomic placement in this species group (Fooden, 1980). However, the two‐way intersection between *M*. *arctoides* and *M*. *thibetana* had the fewest shared heterozygous sites, which is likely due to the low overall heterozygosity in the *M*. *thibetana* sample (Figure 2c; Table 1). *M*. *assamensis* had the largest number of heterozygous sites (~4.7 million; Table 1; Figure 2c).

### 3.1 Extensive Mosaicism of the *M. arctoides* Genome

**FIGURE 3:**
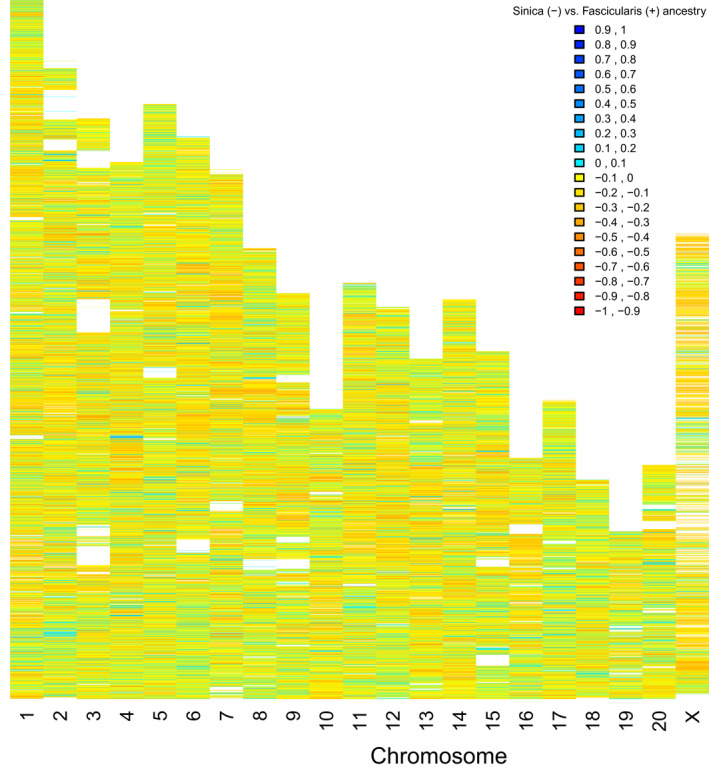
Introgression analysis. Results from analysis of introgression (*f_dM_*) between *M*. *arctoides* samples and the parent taxa in 50 kb sliding windows. Regions where *M*. *arctoides* has most recent ancestry with the *sinica* group are displayed as negative values and regions where *M*. *arctoides* shares the most recent common ancestry with the *fascicularis* group are displayed as positive values. Other window size results are in Figure S3 and plots using individual *M*. *arctoides* samples are in Figure S4

The four‐taxon test (Green et al., 2010; Kulathinal et al., 2009; Martin et al., 2015) results which used all three *M*. *arctoides* samples, supported that the *M*. *arctoides* genome has a mosaic of shared ancestry with both the *sinica* and *fascicularis* species groups (Figure 3). While this result is consistent with extensive introgression from both species groups, this pattern is also consistent with incomplete lineage sorting (ILS) between these taxa (Edelman et al., 2019; Stevison & Kohn, 2009) (See Supplementary Materials). The genome‐wide average *f_dM_* (Malinsky et al., 2015; Martin, 2019) value was −0.113, ranging from −0.69 (with negative values supporting shared ancestry with the *sinica* group) to 0.55 (with positive values supporting shared ancestry with the *fascicularis* group) in 50 kb sliding windows. This sliding window analysis was repeated with several window sizes (Figure S3), and all had a similar genome‐wide mean *f_dM_*. In a previous study, estimates of Patterson's *D* (Green et al., 2010) were similar to the *f_dM_* estimates here (see Table S8 in (Fan et al., 2018)).

Phylogenetic relationships among the species groups were evaluated via the software Twisst (Martin & Belleghem, 2017). This method quantifies phylogenetic relationships across the genome and returns weights in sliding windows (Figure 2d). The topology weights were used to define a “majority topology” as having two‐thirds or more of the sum of weights in any region. Windows that did not meet this criterion were re‐classified as “unresolved.” This analysis showed the proportion of the genome that supported “topo2” (fas,(arc,sin)), which groups *M*. *arctoides* with the *sinica* group, as 52.64% (95% bootstrap CI [52.02%, 53.28%]). However, a significant portion, 15.70% (95% bootstrap CI [15.40%, 16.01%]), of the genome supported “topo3” ((arc,fas),sin), which groups *M*. *arctoides* with the *fascicularis* species group. Interestingly, 11.07% (95% bootstrap CI [10.84%, 11.31%]) of the genome supported *M*. *arctoides* clustering in a group by itself outside of the *sinica* and *fascicularis* groups (“topo1”; (arc,(fas,sin))). The remaining 20.59% (95% bootstrap CI [20.18%, 21.01%]) of the genome was categorized as unresolved because none of the three topologies had >2/3^rds^ of weight. That the 95% confidence intervals of topo3 do not overlap with those of topo1 is consistent with the putative hybrid origin of *M*. *arctoides*, as opposed to topo1 and topo3 both stemming from ancestral polymorphism (but see below).

However, a subsequent investigation of nucleotide divergence of these genomic regions revealed that *D_XY_* between sister taxa in regions supporting topo2 (fas,(arc,sin)) was significantly lower than in regions supporting topo3 ((arc,fas),sin) (Figure S5). Rather than being consistent with HHS, this result is instead consistent with either *M*. *arctoides* being sister to the *fascicularis* group with subsequent hybridization with the *sinica* species group (Figure S1B), or with *M*. *arctoides* splitting from macaques prior to the split of the *sinica* and *fascicularis* species groups with subsequent hybridization from each group at different times (Figure S1C). This latter scenario is consistent with the deepest nucleotide divergence being in regions supporting topo1 (arc(sin,fas)). However, this analysis does not include additional members of the *sinica* species group (e.g., *M*. *sinica* and *M*. *radiata*) that would be crucial to resolving this complex evolutionary history (see Supplementary Materials).

### 3.2 Investigation of outlier genes associated with genital morphology

Our gene‐centered approach led to the calculation of seven population genetic statistics across all genes in the rheMac8 annotation. In total, our analysis focused on 10,543 genes for which we were able to get reliable estimates for each metric, including recombination rate (Table S6). However, for the *dN*/*dS* calculations, this baseline number of genes was far fewer with only 1903 due to the limitation of our approach (see Methods). Of the 2284 genes that were included on our candidate gene list (Table 2 and S4) and also had orthologs in rheMac8, only 1055 were included in our analyses (Table 2 and S6). Of the genome‐wide outliers across these metrics, 67 of these 1055 genes were statistically elevated as potential outliers for further investigation (Table S7).

**FIGURE 4:**
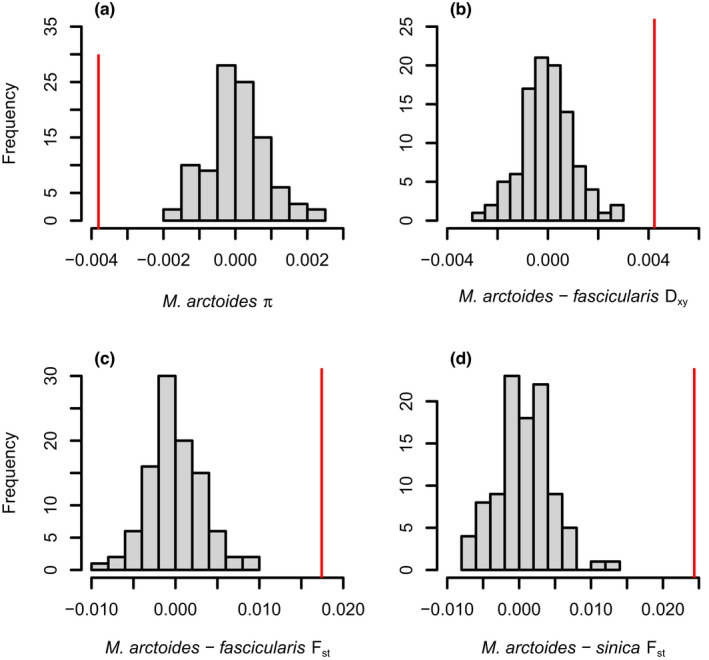
Permutation test results. For each panel, the red line is the difference between the mean of all genital genes and all other genes for a given summary statistic. Additionally, a histogram of the results from 100 permutations where the mean difference was recalculated using the same number of randomly selected genes as the candidate genital genes is shown. The various summary statistics plotted here are (a) *π* in *M*. *arctoides*, (b) *D_XY_* between *M*. *arctoides* and *fascicularis* group species, (c) *F*_ST_ between *M*. *arctoides* and *fascicularis* group species, and (d) *F*_ST_ between *M*. *arctoides* and *sinica* group species. The computed p‐values for all tests shown here were zero. The remaining statistics were not significantly different and are reported in Table S6

A chi‐square test was used to compare the proportion of outlier candidate genes (OCGs) against those analyzed to the expected number of outliers based on the remainder of the genes in the genome (Table S6). This analysis revealed that there were no more outliers than expected by chance, suggesting that the 67 outliers are not more than would be expected of any other set of candidate genes. However, a permutation test was used to compare the difference between means across the seven population genetic metrics found that for four metrics, the mean of the candidate genes was significantly different than expected based on the value across the remainder of the genes in the genome (Figure 4). These metrics showed that the nucleotide diversity, *π*, across the candidate genes was significantly lower than expected (Figure 4a). Additionally, both measures of *F_ST_* between *M*. *arctoides* and the two species groups revealed higher than expected nucleotide divergence (Figure 4c,d). Similarly, *D_XY_* between *M*. *arctoides* and the *fascicularis* species group was much higher than expected based on 100 permutations of a similar number of genes that were not part of the candidate gene set (Figure 4b).

An investigation into the 67 genes found that three genes were OCGs across four statistics (CEP19, GATA3, and PTPN23). Three more genes were OCGs across three statistics (AIRE, CSPP1, and SNAI2). Finally, 11 genes were OCGs across two of the seven statistics (CAMKV, DND1, HMGA1, HSPA4, KDM3B, PIK3CA, PSMB8, SIN3A, TDRKH, TMED10, and ZCWPW1). It is worth noting that although the genes on the HPO and MP database lists were under the specific search terms, each of these genes was associated with many unrelated phenotypes, thus it is difficult to interpret their overall significance in this analysis. For OCGs from the HPO database, each gene had an average of 70.8 phenotypic terms with a range from 4 to 302. For the OCGs in the mammalian phenotype database, the average number of phenotypic terms was smaller at 28.2, and the range was from 1 to 190. This suggests the mammalian database may be slightly better curated to avoid genes that are overly pleiotropic. Interestingly, of these 17 OCGs that were outliers across more than one statistic, only two have putative high impact variants (CEP19 and CSPP1), with the latter also having 15 missense mutations of putative moderate impact. CSPP1 is associated with 151 HPO terms, being presumably involved in a lot of different phenotypes (Table S7).

Despite the uncertainty of hits in the larger databases, seven of the OCGs were on the original literature survey for candidate genes (Table 2 and S4), which were more carefully selected based on their role in genital morphology. Of these, GATA3 was an outlier across multiple statistics, which was initially included due to its known association with urogenital anomalies (Délot et al., 2017), with over 20 years of research on this gene (Lemos & Thakker, 2020), including knockout studies (Grote et al., 2008). Another of these seven is HFE, which is associated with hereditary hemochromatosis (HH) and subsequent hypogonadism (Délot et al., 2017) due to decreased iron circulation (Bezwoda et al., 1977; McDermott & Walsh, 2005), with symptoms such as decreased libido and testicular atrophy in humans (Kelly et al., 1984). In our SNP effect analysis, GATA3 had 1 predicted moderate impact variant and 8 low impact variants (Table S7).

**FIGURE 5:**
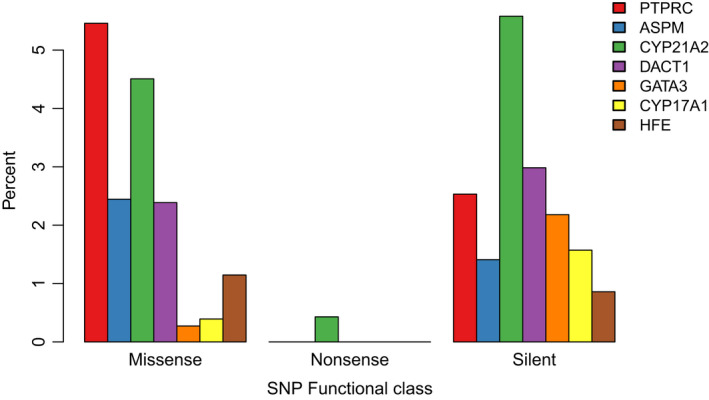
Functional impact of variants in outlier candidate genes. Results from SNPeff are shown broken down by functional impact on the protein sequence of the seven outlier candidate genes based on the literature list. Functional classes are missense variants that change the amino acid, nonsense, which results in a premature stop codon, and silent variants, which alter the nucleotide sequence but result in synonymous amino acid

Additionally, two of the seven OCGs from the literature survey gene list were CYP proteins (CYP17A1 and CYP21A2). Both CYP17A1 and CYP21A2 are associated with congenital adrenal hyperplasia (CAH aka 17OHD), a disorder that leads to abnormal sexual development (Délot et al., 2017), with CYP21A2 mutations being the most common cause of CAH (Koppens et al., 1992). CYP21A2 mutations in humans have been associated with simple virilization, in which female genitalia are masculinized during development (Koppens et al., 1992). Several cohort studies have found genotype‐to‐phenotype associations linking CYP21A2 mutations to a variety of CAH phenotypes (de Carvalho et al., 2016; Soardi et al., 2008; Torres et al., 2003). Additionally, protein structure analysis has been done to examine the impact of specific mutants on a form of CAH known as the salt‐water wasting disease, which if left undiagnosed leads to low sodium levels and death (Haider et al., 2013). CYP17A1 is heavily involved in steroidogenesis, with deletion mutations leading to hormone production inhibition and sex development abnormalities (Keskin et al., 2015; Papi et al., 2018; Turkkahraman et al., 2015; Xia et al., 2021; Zhang et al., 2015). Recent genome studies of 17OHD patients revealed 46,XY karyotypes in physically presenting females which co‐occur with multiple mutations in the CYP17A1 gene (Xia et al., 2021; Zhang et al., 2015). CYP21A1 had two high, 21 moderate, and 32 low impact variants, whereas CYP17A1 had two moderate and eleven low impact variants (Table S7). Additionally, CYP21A1 had the highest proportion of silent and nonsense variants, as well as the 2^nd^ highest proportion of missense variants (Figure 5).

Three more OCGs on the original literature candidate gene list that were outliers for *dN*/*dS* were identified in a study as major candidates for baculum morphology in mice (Schultz, Ingels, et al., 2016) (DACT1, PTPRC, and ASPM; Figure 5). This is particularly intriguing when one considers the unique baculum morphology of the *os penis* in the bear macaque relative to all other primates (see Discussion). Because these were outliers for *dN*/*dS*, which is targeted to the protein‐coding regions of the gene, we further investigated the protein sequence of each gene to look at coding mutations. PTPRC had the highest proportion of missense mutation among the seven literature outliers (Figure 5). Specifically, there were seven amino acid changes in multiple individuals, two of which overlapped the fibronectin domain and had significant changes in the amino acid properties. Two of these changes were completely fixed in *M*. *arctoides* (p.K449E and p.R424R), two were nearly fixed, shared only with a single *M*. *assamensis* sample (p.E314G and p.Y359S), and three amino acid changes were shared between *M*. *arctoides* and members of the *fascicularis* species group (p.D363G, p.K384N, and p.D385N). DACT1 had the 2^nd^ highest proportion of silent variants among the seven literature OCGs (Figure 5). Additionally, we found 3 major amino acid changes clustered together, two that were nearly fixed in *M*. *arctoides* as compared to all other macaques (p.C439Y and p.G493D). Additionally, there was a p.Q498R mutation that was shared between *M*. *arctoides* and members of the *fascicularis* species group. This similarity is of particular interest due to the shared baculum morphology of these species (see Discussion). Finally, for ASPM, there was only one fixed amino acid change (p.H352P), which overlapped a region of the protein that interacts with another enzyme called katanin. Additionally, there was a p.H1870R mutation that was shared with *sinica* species, and several other amino acid changes that were unique to *M*. *arctoides*, but polymorphic rather than fixed (p.V269F, p.T357I, and p.R1164H). While all of these amino acid changes are likely to have an impact on the protein, the specific changes to the gene function and its overall implications for the baculum phenotype are unknown. Still, these residues would be of particular interest in mouse mutant studies on the genetics of baculum morphology.

Permutation analysis of male versus female genital development genes showed no significant difference in all statistics except *f_dM_* (*p* = 0.02), where male genital genes exhibited higher introgression between *M*. *arctoides* and *sinica* species than female genes (Table S5).

## 4 DISCUSSION

Genital morphology is an important trait that may evolve rapidly. In the bear macaque, the divergent genital morphology appears to be consistent with coevolution between males and females following a “lock‐and‐key” mechanism. We examined the intersection of genome‐wide outliers in several population genetic metrics with genes associated with genital morphology in mammals to identify genes that might explain the unique genital evolution of the bear macaque.

### 4.1 Genital morphology genes have higher divergence and lower diversity than expected

Although the number of outliers from our genital candidate genes was not more than expected by chance, the average divergence of these genes was higher than expected and the diversity was lower than expected (Figure 4). None of the permutations overlapped the observed mean difference between the actual candidate gene set for four of the seven population genetic metrics. Specifically, the candidate genes had lower *π* which is consistent with recent positive selection on these genes that would have reduced local genetic variation. Similarly, *F_ST_* between *M*. *arctoides* and both species groups was higher than expected, which is also consistent with positive selection on this subset of genes as compared to the remainder of the genome. Additionally, *D_XY_* between *M*. *arctoide*s and *fascicularis* species was elevated, but not *D_XY_* between *M*. *arctoides* and *sinica* species. These results suggest that the rate of evolution of these genes is higher than the rest of the genes in the genome, confirming the importance of genital morphology in the evolution of this species. A possible explanation for the difference in the two measures of nucleotide divergence is that *F_ST_* is a relative measure of divergence, whereas *D_XY_* is an absolute measure (Noor & Bennett, 2009). Here, the majority of the genome clusters with *sinica* species, which could explain why *D_XY_* between *M*. *arctoide*s and the *sinica* species group does not have more outliers than expected.

Of 67 candidate genes identified as outliers, many are involved in a variety of phenotypes. Of course, because these are developmental phenotypes, it is not surprising that the functions of these genes would be highly pleiotropic. However, their specific role in contributing to the divergent genital morphology and subsequent reproductive isolation in this species is unclear. Of note, the number of genes in both phenotype ontology databases associated with male genital abnormalities is nearly 2x the number of genes associated with female genital abnormalities (Table 2). This is not likely due to underlying differences in the genetics of these traits, but instead more likely reflects a bias toward research into male reproductive morphology as compared to female reproductive abnormalities (Sloan & Simmons, 2019). Still, for the most part, the mean values across our various metrics were not significantly different between these sets of male vs. female genes (Table S5). While the result for *f_dM_* was statistically significant, both values were lower than the genome‐wide mean, and thus do not likely reflect differences in the direction of introgression. In contrast, the difference in means of *dN*/*dS*, though not statistically significant, had twice the difference of *f_dM_*. Specifically, the mean *dN*/*dS* across male genes (0.141) matches the mean of the genome background (0.143), whereas the mean across female genes was much lower (0.101). Though speculative, this difference is perhaps consistent with the idea that many genes of large effect on female genital morphology have yet to be described, supported by the disparity in the gene set sizes. If the *dN*/*dS* difference were to remain the same after more female genital morphology genes have been described, it would suggest male genitalia evolve at a more rapid rate than female genitalia, in support of sexual conflict. However, given that the variety of divergence statistics used in this study does not have an apparent difference between male and female genital morphology genes, it seems likely that this is not the case and instead male and female genitalia in *M*. *arctoides* coevolve together. Together these findings suggest that future work should aim to identify additional potentially undiscovered genes that play a role in female genital morphology.

### 4.2 Divergent baculum morphology putatively associated with *dN/dS* outliers

Regardless of the inconclusiveness of the genes that were found based on phenotype ontology methods, 3/21 outliers for *dN*/*dS* were genes previously identified in mice as candidate genes for morphological variation in the baculum (DACT1, PTPRC, and ASPM). The baculum, or penis bone, is a novel male internal genital structure in placental mammals that evolved ~145 million years ago (Brindle & Opie, 2016), with the bear macaque, having the longest primate baculum. Since that time, it has been gained or lost multiple times, including a recent loss in *Homo sapiens* (Carosi & Scalici, 2017; Schultz et al., 2016). Despite its remarkable interspecific variation, the penis bone generally does not vary extensively within species (Ramm et al., 2009), and maybe the only physical trait that differs consistently between closely related species, making it a key morphological character for species identification (Carosi & Scalici, 2017). However, the genetics of morphological variation in this trait is only beginning to be understood. In 2016, a morphometric analysis using micro‐CT scans of bacula in mice identified three major quantitative trait loci, two that together explained 50.6% of the variation in baculum size, and one that explained 23.4% of the variation in baculum shape (Schultz, Ingels, et al., 2016). These regions were further narrowed by both differential gene expression in the early development of male mice, and potential involvement in bone or genital morphogenesis, revealing 16 major candidate loci with potential effects on baculum development and morphology (see table S3 in Schultz, Ingels, et al., 2016), that were included on our candidate gene list (Table S4).

The baculum in *M*. *arctoides* is more than double the average length of *sinica* species group taxa and nearly four times as long as *fascicularis* species group taxa (Dixson, 1987; Fooden, 1971). In addition to its elongation, the morphology of the *M*. *arctoides* baculum also has a unique arced crescent shape, with an absent distal region that is present in most other macaque species (See the comparison in figure 8 of Fooden et al., 1985; Fooden, 1990). Among macaques, these two features—a gentle dorsoventral curvature and the lack of a well‐defined distal process—are shared only by members of the *fascicularis* species group. This latter point perhaps explains why 4/5 amino acid changes across these three genes that were shared between *M*. *arctoides* and another group, were shared between *M*. *arctoides* and the *fascicularis* species members. This is in stark contrast to the rest of the genome which has a much higher proportion of shared variation with *sinica* species group members.

### 4.3 Future directions

This study has focused on genes with phenotypic associations with genital morphology, but an outlier analysis simply presents hypotheses of the specific genes involved in the divergent reproductive traits of the bear macaque. A target of future functional work should be to identify their specific role (if any) in contributing to either divergent male genital morphology or compensatory changes in female reproductive morphology. They should also be the target of functional studies in model systems, such as mice, on the genital morphology of both males and females. For example, the seven fixed amino acid changes in the bear macaque across the three *dN*/*dS* outlier genes associated with mouse baculum morphology could be prioritized in future work on the genetics of this trait. Still, why this remarkable trait is divergent in the bear macaque is unknown. Perhaps more detailed functional connections between specific genes/variants and the baculum would allow for future work to examine the selective processes that shaped this unique genital phenotype.

Additionally, as the genetic basis of other genital traits, such as baubellum morphology becomes known, the candidate gene list can be extended. Similarly, as the genome annotation improves with subsequent reference genomes, more genes can be considered that have clear orthologs in macaques.

In addition to this study, there has been extensive interest in the evolution of this species. However, due to the limited number of publicly available whole‐genome samples (now three with the present study), the conservation status of this species, and the ethical and logistical barriers to experimental research with primates, it is challenging to make functional insights into its evolution. Here, our results provide conflicting support both for and against an HHS scenario, proposing new alternatives that can be addressed in future studies on the evolution of this species. Additional WGS data from more individuals of this species would allow for a better characterization of selection/adaptation within this lineage. Additionally, more WGS samples would be useful in examining the regions of excess shared ancestry for more direct tests that rule out ILS so that hybridization can be better quantified and understood.

## AUTHOR CONTRIBUTIONS

**Laurie S. Stevison:** Conceptualization (equal); Data curation (lead); Formal analysis (lead); Investigation (lead); Methodology (lead); Project administration (equal); Writing – original draft (lead); Writing – review & editing (lead). **Nick P. Bailey:** Formal analysis (supporting); Investigation (supporting); Methodology (supporting); Writing – original draft (supporting); Writing – review & editing (supporting). **Zachary A. Szpiech:** Formal analysis (supporting); Investigation (supporting); Methodology (supporting); Writing – original draft (supporting); Writing – review & editing (supporting). **Taylor E. Novak:** Formal analysis (supporting); Investigation (supporting); Methodology (supporting); Writing – original draft (supporting); Writing – review & editing (supporting). **Don J. Melnick:** Conceptualization (equal); Project administration (equal); Resources (lead). **Ben J. Evans:** Conceptualization (equal); Formal analysis (equal); Investigation (equal); Methodology (equal); Project administration (equal); Resources (supporting); Software (equal); Supervision (equal); Writing – original draft (equal); Writing – review & editing (equal). **Jeffrey D. Wall:** Conceptualization (equal); Funding acquisition (lead); Investigation (equal); Project administration (equal); Supervision (equal); Writing – original draft (supporting).

### OPEN RESEARCH BADGES

This article has earned an Open Data Badge for making publicly available the digitally‐shareable data necessary to reproduce the reported results. The data is available at https://doi.org/10.5281/zenodo.5006971 and http://dx.doi.org/10.35099/aurora‐67.

## Supporting information

Tables S1–S7

Supplementary Material

## Data Availability

Raw read data from two sequenced samples have been deposited in the NCBI sequence read archive (PRJNA622565). Additionally, all code and a detailed workflow of the commands used are provided in a GitHub repository (https://github.com/StevisonLab/Arctoides‐Hybridization). A snapshot of the code at the time of publication is available at https://doi.org/10.5281/zenodo.6459172. The resulting VCF files have been deposited in the institutional repository Aurora (http://dx.doi.org/10.35099/aurora‐67).
