# Supplementary material for: Evolution of genes involved in the unusual genitals of the bear macaque, Macaca arctoides
Source: Ecol Evol. 2022 May 24;12(5):e8897. doi: 10.1002/ece3.8897 (PMC9130562; doi:10.1002/ece3.8897)
Supplement: Supplementary file 1 — Tables S1–S7 [file ECE3-12-e8897-s002.docx]

**Table S1.** Summary statistics of each sample throughout the genome analysis pipeline.

| **Sample ID** | **Read Count** | **Read Length** | **Percentage Mapped** | **Percentage Duplicated** | **Percentage unique variants BQSR Step 1** | **Percentage unique variants BQSR Step 2** | **VCF Coverage** | **VCF Coverage Standard Deviation** |
| --- | --- | --- | --- | --- | --- | --- | --- | --- |
| XH1 | 1,231,654,664 | 126 | 62.42% | 8.93% | 1.90% | 0.10% | 47.2136 | 36.0059 |
| SM1 | 1,001,034,260 | 101 | 60.73% | 6.93% | 1.60% | 0.00% | 31.1149 | 23.3435 |
| SM2 | 471,805,366 | 126 | 66.82% | 8.96% | 1.60% | 0.00% | 19.1474 | 19.4368 |
| CR-5 | 3,299,851,568 | 45 | 52.90% | 11.71% | 2.60% | 0.00% | 43.4295 | 30.5846 |
| BGI | 706,459,956 | 50 | 53.54% | 8.44% | 2.60% | 0.00% | 8.66336 | 13.9151 |
| BGI-96346 | 311,203,360 | 45 | 52.06% | 6.53% | 2.60% | 0.00% | 4.02831 | 12.8233 |
| BGI-CE-4 | 3,692,987,634 | 45 | 52.38% | 17.22% | 0.80% | 0.00% | 32.0124 | 20.8328 |
| Malaya | 656,978,010 | 101 | 62.39% | 8.24% | 1.60% | 0.00% | 19.138 | 19.8059 |
| A20 | 834,133,746 | 101 | 46.23% | 7.84% | 1.90% | 0.10% | 13.3644 | 27.2626 |
| Tibetan-macaque-NO.3 | 1,287,914,270 | 101 | 60.57% | 10.28% | 2.40% | 0.00% | 25.6008 | 16.3121 |
| PF660 | 953,794,267 | 302 | 70.84% | 12.33% | 2.10% | 0.00% | 39.0705 | 31.7348 |
| PM664 | 1,004,916,756 | 302 | 72.45% | 13.33% | 1.90% | 0.00% | 40.486 | 40.2966 |
| PM592 | 968,791,330 | 302 | 72.37% | 13.15% | 2.10% | 0.00% | 38.6892 | 37.1028 |

**Table S2.** File information for data accessed via mGAP. We merged the following files from mGAP and Liu et al 2018 and restricted only to biallelic SNPs. The sites we obtain from Liu et al. (2018) form a training set of slightly less confident sites, while the mGAP sites formed a set of our most confident polymorphic sites. The mGAP database not only follows GATK best practices to call variants, but also exploits known pedigrees to check for Mendelian inconsistencies.

| **File Name** | **Sample Size** | **Sampling Location** | **Animal Type** | **Data Source** |
| --- | --- | --- | --- | --- |
| CPRC_Animals.vcf.gz | 10 | Caribbean Primate Research Center (CPRC) | Indian Macaque | mGAP, Xue et al. (2016) |
| CNPRC_Animals.vcf.gz | 17 | California National Primate Research Center (CNPRC) | Indian Macaque | mGAP, Xue et al. (2016) |
| mGap.v1.7.vcf.gz | 293 | Oregon National Primate Research Center (ONPRC) | Indian Macaque | mGAP v1.7 |
| NEPRC_Animals.vcf.gz | 12 | New England Primate Research Center (NEPRC) | Indian Macaque | mGAP, Xue et al. (2016) |
| SNPRC_Animals.vcf.gz | 2 | Southwest National Primate Research Center (SNPRC) | Indian Macaque | mGAP, Xue et al. (2016) |
| TNPRC_Animals.vcf.gz | 10 | Tulane National Primate Research Center (TNPRC) | Indian Macaque | mGAP, Xue et al. (2016) |
| WNPRC_Animals.vcf.gz | 32 | Wisconsin National Primate Research Center (WNPRC) | Indian Macaque | mGAP, Xue et al. (2016) |
| YNPRC_Animals.vcf.gz | 13 | Yerkes National Primate Research Center (YNPRC) | Indian Macaque | mGAP, Xue et al. (2016) |
| 81wildChineseRhesus.vcf.gz | 81 | Multiple Locations in China | Chinese Macaque | Liu et al. (2018) |

**Table S3.** Introgression test results for different combinations of sample sets and inviduals. For each comparison, the average number of sites used per 50kb window is reported.

Additionally, the mean and standard deviation for D is reported. fd is a more conservative estimator for the ABBA-BABA test. However, if D is negative, fd becomes meaningless.

Alternatively, the fdM statistic can be used to report either an excess of shared variation between P1 and P3 (negative D, negative fdM) or P2 and P3 (positive D, positive fdM). See github.com/simonhmartin/genomics_general for full explanation.

| **P1** | **P2** | **P3** | **Avg # sitedUsed** | **D** | **stdev** | **fd** | **fdM** | **stdev3** |
| --- | --- | --- | --- | --- | --- | --- | --- | --- |
| **SIN** | **FAS** | **ARC** | 259.363 | -0.459 | 0.62 | n/a | -0.113 | 0.16 |
| SIN | *M. mulatta* | ARC | 225.04 | -0.453 | 0.62 | n/a | -0.104 | 0.15 |
| SIN | *M. fascicularis* | ARC | 210.858 | -0.473 | 0.62 | n/a | -0.104 | 0.14 |
| *M. thibetana* | FAS | ARC | 210.025 | -0.467 | 0.64 | n/a | -0.101 | 0.15 |
| *M. assamensis* | FAS | ARC | 242.915 | -0.454 | 0.62 | n/a | -0.110 | 0.16 |

**Table S4.** List of 141 candidate genes from comprehensive literature search. Note, 31 genes do not have orthologys in RheMac8, meaning that only 110 were considered moving forward. Acronyms: DSD="Disordered Sex Development"; GT="Genital Tubercle".

| **Gene Name** | **Organism** | **RheMac8 Ortho** | **Reference** | **Function** |
| --- | --- | --- | --- | --- |
| AKR1C | Horse/Human | NA | https://[www.mdpi.com/2073-4425/11/3/251](http://www.mdpi.com/2073-4425/11/3/251) | Affects steroid hormones; mutations associated with DSD |
| AKR1C2 | Human | NA | https://[www.ncbi.nlm.nih.gov/pmc/articles/PMC5714504/](http://www.ncbi.nlm.nih.gov/pmc/articles/PMC5714504/) | Sex differentiation, part of primary gene list used at the UCLA… |
| AKR1C4 | Human | ENSMMUG00000000958.3 | https://[www.ncbi.nlm.nih.gov/pmc/articles/PMC5714504/](http://www.ncbi.nlm.nih.gov/pmc/articles/PMC5714504/) | Sex differentiation, part of primary gene list used at the UCLA… |
| AMH | Human | NA | https://[www.ncbi.nlm.nih.gov/pmc/articles/PMC5714504/](http://www.ncbi.nlm.nih.gov/pmc/articles/PMC5714504/) | Sex differentiation, part of primary gene list used at the UCLA… |
| AMHR2 | Human | ENSMMUG00000020037.3 | https://[www.ncbi.nlm.nih.gov/pmc/articles/PMC5714504/](http://www.ncbi.nlm.nih.gov/pmc/articles/PMC5714504/) | Sex differentiation, part of primary gene list used at the UCLA… |
| APC | Mouse | ENSMMUG00000000330.3 | https://academic.oup.com/biolreprod/article/102/6/1248/5811995 | Downregulated when females exposed to masculinizing substance |
| AR | Human | ENSMMUG00000011437.3 | https://[www.ncbi.nlm.nih.gov/pmc/articles/PMC5714504/](http://www.ncbi.nlm.nih.gov/pmc/articles/PMC5714504/) | Sex differentiation, part of primary gene list used at the UCLA… |
| ARF6 | Mouse | ENSMMUG00000041889.1 | https://[www.ncbi.nlm.nih.gov/pmc/articles/PMC4856068/](http://www.ncbi.nlm.nih.gov/pmc/articles/PMC4856068/) | Osteoclast activity |
| ARL6 | Human | ENSMMUG00000038349.1 | https://[www.ncbi.nlm.nih.gov/pmc/articles/PMC5714504/](http://www.ncbi.nlm.nih.gov/pmc/articles/PMC5714504/) | Hypogonadism, part of primary gene list used at the UCLA… |
| ARX | Human | NA | https://[www.ncbi.nlm.nih.gov/pmc/articles/PMC5714504/](http://www.ncbi.nlm.nih.gov/pmc/articles/PMC5714504/) | Sex differentiation, part of primary gene list used at the UCLA… |
| ASPM | Mouse | ENSMMUG00000000245.3 | https://[www.ncbi.nlm.nih.gov/pmc/articles/PMC4856068/](http://www.ncbi.nlm.nih.gov/pmc/articles/PMC4856068/) | Genes associated with bone size/density and part of QTL's associated with baculum variation |
| ATRX | Human | ENSMMUG00000022574.3 | https://[www.ncbi.nlm.nih.gov/pmc/articles/PMC5714504/](http://www.ncbi.nlm.nih.gov/pmc/articles/PMC5714504/) | Sex differentiation, part of primary gene list used at the UCLA… |
| BBS1 | Human | NA | https://[www.ncbi.nlm.nih.gov/pmc/articles/PMC5714504/](http://www.ncbi.nlm.nih.gov/pmc/articles/PMC5714504/) | Hypogonadism, part of primary gene list used at the UCLA… |
| BBS10 | Human | NA | https://[www.ncbi.nlm.nih.gov/pmc/articles/PMC5714504/](http://www.ncbi.nlm.nih.gov/pmc/articles/PMC5714504/) | Hypogonadism, part of primary gene list used at the UCLA… |
| BBS12 | Human | ENSMMUG00000030171.2 | https://[www.ncbi.nlm.nih.gov/pmc/articles/PMC5714504/](http://www.ncbi.nlm.nih.gov/pmc/articles/PMC5714504/) | Hypogonadism, part of primary gene list used at the UCLA… |
| BBS2 | Human | ENSMMUG00000013268.3 | https://[www.ncbi.nlm.nih.gov/pmc/articles/PMC5714504/](http://www.ncbi.nlm.nih.gov/pmc/articles/PMC5714504/) | Hypogonadism, part of primary gene list used at the UCLA… |
| BBS4 | Human | ENSMMUG00000013685.3 | https://[www.ncbi.nlm.nih.gov/pmc/articles/PMC5714504/](http://www.ncbi.nlm.nih.gov/pmc/articles/PMC5714504/) | Hypogonadism, part of primary gene list used at the UCLA… |
| BBS5 | Human | NA | https://[www.ncbi.nlm.nih.gov/pmc/articles/PMC5714504/](http://www.ncbi.nlm.nih.gov/pmc/articles/PMC5714504/) | Hypogonadism, part of primary gene list used at the UCLA… |
| BBS7 | Human | ENSMMUG00000009819.3 | https://[www.ncbi.nlm.nih.gov/pmc/articles/PMC5714504/](http://www.ncbi.nlm.nih.gov/pmc/articles/PMC5714504/) | Hypogonadism, part of primary gene list used at the UCLA… |
| BBS9 | Human | ENSMMUG00000002886.3 | https://[www.ncbi.nlm.nih.gov/pmc/articles/PMC5714504/](http://www.ncbi.nlm.nih.gov/pmc/articles/PMC5714504/) | Hypogonadism, part of primary gene list used at the UCLA… |
| BCL9 | Mouse | ENSMMUG00000016786.3 | https://academic.oup.com/biolreprod/article/102/6/1248/5811995 | Upregulated when female exposed to masculinizing substance |
| BMP15 | Human | ENSMMUG00000017358.2 | https://[www.ncbi.nlm.nih.gov/pmc/articles/PMC5714504/](http://www.ncbi.nlm.nih.gov/pmc/articles/PMC5714504/) | Sex determination, part of primary gene list used at the UCLA Clinical Genome Center to call DSD variants |
| BMP4 | Mouse | ENSMMUG00000000429.3 | https://dev.biologists.org/content/develop/127/11/2471.full.pdf | expressed in the distal urethral plate epithelium of the GT |
| CBX2 | Human | ENSMMUG00000046417.1 | https://[www.ncbi.nlm.nih.gov/pmc/articles/PMC5714504/](http://www.ncbi.nlm.nih.gov/pmc/articles/PMC5714504/) | Sex determination, part of primary gene list used at the UCLA Clinical Genome Center to call DSD variants |
| CCND3 | Mouse | ENSMMUG00000004419.3 | https://academic.oup.com/biolreprod/article/102/6/1248/5811995 | Upregulated when female exposed to masculinizing substance |
| CFHR2 | Mouse | NA | https://[www.ncbi.nlm.nih.gov/pmc/articles/PMC4856068/](http://www.ncbi.nlm.nih.gov/pmc/articles/PMC4856068/) | Regulation of bone mass |
| CHD7 | Human | ENSMMUG00000011584.3 | https://[www.ncbi.nlm.nih.gov/pmc/articles/PMC5714504/](http://www.ncbi.nlm.nih.gov/pmc/articles/PMC5714504/) | Hypogonadism, part of primary gene list used at the UCLA… |
| CSNK1A1 | Mouse | ENSMMUG00000001758.3 | https://academic.oup.com/biolreprod/article/102/6/1248/5811995 | Upregulated when female exposed to masculinizing substance |
| CSNK1D | Mouse | ENSMMUG00000018329.3 | https://academic.oup.com/biolreprod/article/102/6/1248/5811995 | Downregulated when females exposed to masculinizing substance |
| CTNNB1 | Mouse | ENSMMUG00000014627.3 | https://academic.oup.com/biolreprod/article/102/6/1248/5811995 | Upregulated when female exposed to masculinizing substance |
| CYP11A1 | Human | ENSMMUG00000003239.3 | https://[www.ncbi.nlm.nih.gov/pmc/articles/PMC5714504/](http://www.ncbi.nlm.nih.gov/pmc/articles/PMC5714504/) | Sex differentiation, part of primary gene list used at the UCLA… |
| CYP11B1 | Human | ENSMMUG00000013631.3 | https://[www.ncbi.nlm.nih.gov/pmc/articles/PMC5866176/](http://www.ncbi.nlm.nih.gov/pmc/articles/PMC5866176/) | Mutations associated with disorders of steroidogenesis associated with 46,XX DSD |
| CYP17A1 | Human | ENSMMUG00000017601.3 | https://[www.ncbi.nlm.nih.gov/pmc/articles/PMC5714504/](http://www.ncbi.nlm.nih.gov/pmc/articles/PMC5714504/) | Sex differentiation, part of primary gene list used at the UCLA… |
| CYP19A1 | Human | ENSMMUG00000002553.3 | https://[www.ncbi.nlm.nih.gov/pmc/articles/PMC5714504/](http://www.ncbi.nlm.nih.gov/pmc/articles/PMC5714504/) | Sex differentiation, part of primary gene list used at the UCLA… |
| CYP21A2 | Human | ENSMMUG00000000755.3 | https://[www.ncbi.nlm.nih.gov/pmc/articles/PMC5714504/](http://www.ncbi.nlm.nih.gov/pmc/articles/PMC5714504/) | Sex differentiation, part of primary gene list used at the UCLA… |
| DACT1 | Mouse | ENSMMUG00000001090.3 | https://[www.ncbi.nlm.nih.gov/pmc/articles/PMC4856068/](http://www.ncbi.nlm.nih.gov/pmc/articles/PMC4856068/) | Genes associated with bone size/density and part of QTL's associated with baculum variation |
| DHCR7 | Human | ENSMMUG00000011976.3 | https://[www.ncbi.nlm.nih.gov/pmc/articles/PMC5714504/](http://www.ncbi.nlm.nih.gov/pmc/articles/PMC5714504/) | Sex differentiation, part of primary gene list used at the UCLA… |
| DHH | Human | ENSMMUG00000012776.3 | https://[www.ncbi.nlm.nih.gov/pmc/articles/PMC5714504/](http://www.ncbi.nlm.nih.gov/pmc/articles/PMC5714504/) | Sex determination, part of primary gene list used at the UCLA Clinical Genome Center to call DSD variants |
| DKK1 | Mouse | ENSMMUG00000010847.3 | https://academic.oup.com/biolreprod/article/102/6/1248/5811995 | Downregulated when females exposed to masculinizing substance |
| DMRT1 | Human | ENSMMUG00000044083.1 | https://[www.ncbi.nlm.nih.gov/pmc/articles/PMC5714504/](http://www.ncbi.nlm.nih.gov/pmc/articles/PMC5714504/) | Sex determination, part of primary gene list used at the UCLA Clinical Genome Center to call DSD variants |
| DMRT2 | Human | ENSMMUG00000014844.3 | https://[www.ncbi.nlm.nih.gov/pmc/articles/PMC5714504/](http://www.ncbi.nlm.nih.gov/pmc/articles/PMC5714504/) | Sex determination, part of primary gene list used at the UCLA Clinical Genome Center to call DSD variants |
| DVL1 | Mouse | ENSMMUG00000007109.3 | https://academic.oup.com/biolreprod/article/102/6/1248/5811995 | Upregulated when female exposed to masculinizing substance |
| DVL2 | Mouse | NA | https://academic.oup.com/biolreprod/article/102/6/1248/5811995 | Upregulated when female exposed to masculinizing substance |
| EMX2 | Human | ENSMMUG00000012415.3 | https://[www.ncbi.nlm.nih.gov/pmc/articles/PMC5866176/](http://www.ncbi.nlm.nih.gov/pmc/articles/PMC5866176/) | involved in the initial differentiation of the bipotential gonad |
| FBXW2 | Mouse | ENSMMUG00000001646.3 | https://academic.oup.com/biolreprod/article/102/6/1248/5811995 | Downregulated when females exposed to masculinizing substance |
| FGF10 | Mouse | ENSMMUG00000047162.1 | https://dev.biologists.org/content/develop/127/11/2471.full.pdf | KO leads to abnormal external genitalia development |
| FGF8 | Mouse | ENSMMUG00000015771.3 | https://dev.biologists.org/content/develop/127/11/2471.full.pdf | expressed in the distal urethral plate epithelium of the GT |
| FGF8 | Human | ENSMMUG00000015771.3 | https://[www.ncbi.nlm.nih.gov/pmc/articles/PMC5714504/](http://www.ncbi.nlm.nih.gov/pmc/articles/PMC5714504/) | Hypogonadism, part of primary gene list used at the UCLA… |
| FGF9 | Human | ENSMMUG00000009912.3 | https://[www.ncbi.nlm.nih.gov/pmc/articles/PMC5866176/](http://www.ncbi.nlm.nih.gov/pmc/articles/PMC5866176/) | promote maintenance of testicular development |
| FGFR1 | Human | ENSMMUG00000022478.3 | https://[www.ncbi.nlm.nih.gov/pmc/articles/PMC5714504/](http://www.ncbi.nlm.nih.gov/pmc/articles/PMC5714504/) | Hypogonadism, part of primary gene list used at the UCLA… |
| FGFR2 | Human | ENSMMUG00000009594.3 | https://[www.ncbi.nlm.nih.gov/pmc/articles/PMC5714504/](http://www.ncbi.nlm.nih.gov/pmc/articles/PMC5714504/) | Sex differentiation, part of primary gene list used at the UCLA… |
| FOXL2 | Human | NA | https://[www.ncbi.nlm.nih.gov/pmc/articles/PMC5714504/](http://www.ncbi.nlm.nih.gov/pmc/articles/PMC5714504/) | Sex differentiation, part of primary gene list used at the UCLA… |
| FOXN1 | Mouse | ENSMMUG00000021975.2 | https://academic.oup.com/biolreprod/article/102/6/1248/5811995 | Upregulated when female exposed to masculinizing substance |
| FRAS1 | Human | ENSMMUG00000002564.3 | https://[www.ncbi.nlm.nih.gov/pmc/articles/PMC5714504/](http://www.ncbi.nlm.nih.gov/pmc/articles/PMC5714504/) | Hypogonadism, part of primary gene list used at the UCLA… |
| FREM2 | Human | ENSMMUG00000002702.3 | https://[www.ncbi.nlm.nih.gov/pmc/articles/PMC5714504/](http://www.ncbi.nlm.nih.gov/pmc/articles/PMC5714504/) | Hypogonadism, part of primary gene list used at the UCLA… |
| FSHR | Human | ENSMMUG00000017196.3 | https://[www.ncbi.nlm.nih.gov/pmc/articles/PMC5714504/](http://www.ncbi.nlm.nih.gov/pmc/articles/PMC5714504/) | Sex determination, part of primary gene list used at the UCLA Clinical Genome Center to call DSD variants |
| FZD1 | Mouse | NA | https://academic.oup.com/biolreprod/article/102/6/1248/5811995 | Upregulated when female exposed to masculinizing substance |
| FZD2 | Mouse | ENSMMUG00000011038.3 | https://academic.oup.com/biolreprod/article/102/6/1248/5811995 | Upregulated when female exposed to masculinizing substance |
| FZD3 | Mouse | ENSMMUG00000007486.3 | https://academic.oup.com/biolreprod/article/102/6/1248/5811995 | Upregulated when female exposed to masculinizing substance |
| FZD4 | Mouse | ENSMMUG00000004048.2 | https://academic.oup.com/biolreprod/article/102/6/1248/5811995 | Upregulated when female exposed to masculinizing substance |
| FZD6 | Mouse | ENSMMUG00000019399.3 | https://academic.oup.com/biolreprod/article/102/6/1248/5811995 | Upregulated when female exposed to masculinizing substance |
| GATA3 | Human | ENSMMUG00000006944.3 | https://[www.ncbi.nlm.nih.gov/pmc/articles/PMC5866176/](http://www.ncbi.nlm.nih.gov/pmc/articles/PMC5866176/) | uterine anomalies |
| GATA4 | Human | ENSMMUG00000019208.3 | https://[www.ncbi.nlm.nih.gov/pmc/articles/PMC5714504/](http://www.ncbi.nlm.nih.gov/pmc/articles/PMC5714504/) | Sex determination, part of primary gene list used at the UCLA Clinical Genome Center to call DSD variants |
| GNRH1 | Human | ENSMMUG00000029018.2 | https://[www.ncbi.nlm.nih.gov/pmc/articles/PMC5714504/](http://www.ncbi.nlm.nih.gov/pmc/articles/PMC5714504/) | Hypogonadism, part of primary gene list used at the UCLA… |
| GRIP1 | Human | ENSMMUG00000016631.3 | https://[www.ncbi.nlm.nih.gov/pmc/articles/PMC5714504/](http://www.ncbi.nlm.nih.gov/pmc/articles/PMC5714504/) | Hypogonadism, part of primary gene list used at the UCLA… |
| HESX1 | Human | ENSMMUG00000007224.3 | https://[www.ncbi.nlm.nih.gov/pmc/articles/PMC5714504/](http://www.ncbi.nlm.nih.gov/pmc/articles/PMC5714504/) | Hypogonadism, part of primary gene list used at the UCLA… |
| HFE | Human | ENSMMUG00000023733.3 | https://[www.ncbi.nlm.nih.gov/pmc/articles/PMC5714504/](http://www.ncbi.nlm.nih.gov/pmc/articles/PMC5714504/) | Hypogonadism, part of primary gene list used at the UCLA… |
| HHAT | Human | ENSMMUG00000016099.3 | https://[www.ncbi.nlm.nih.gov/pmc/articles/PMC5714504/](http://www.ncbi.nlm.nih.gov/pmc/articles/PMC5714504/) | Sex determination, part of primary gene list used at the UCLA Clinical Genome Center to call DSD variants |
| HIF1A | Mouse | ENSMMUG00000021002.3 | https://[www.ncbi.nlm.nih.gov/pmc/articles/PMC4856068/](http://www.ncbi.nlm.nih.gov/pmc/articles/PMC4856068/) | Genes associated with bone size/density and part of QTL's associated with baculum variation |
| HOXA10 | Human | ENSMMUG00000013459.3 | https://pubmed.ncbi.nlm.nih.gov/23376215/ | Mutations associated with (primarilily) female DSD |
| HOXA13 | Human | ENSMMUG00000013463.3 | https://pubmed.ncbi.nlm.nih.gov/23376215/; https://jmg.bmj.com/content/40/4/e49 | Mutations associated with (mostly) female DSD |

| **Gene Name** | **Organism** | **RheMac8 Ortho** | **Reference** | **Function** |
| --- | --- | --- | --- | --- |
| HOXD13 | Mouse | NA | https://dev.biologists.org/content/develop/127/11/2471.full.pdf | expressed in the distal urethral plate epithelium of the GT |
| HSD17B3 | Human | ENSMMUG00000007990.3 | https://[www.ncbi.nlm.nih.gov/pmc/articles/PMC5714504/](http://www.ncbi.nlm.nih.gov/pmc/articles/PMC5714504/) | Sex differentiation, part of primary gene list used at the UCLA… |
| HSD3B2 | Human | ENSMMUG00000016568.3 | https://[www.ncbi.nlm.nih.gov/pmc/articles/PMC5714504/](http://www.ncbi.nlm.nih.gov/pmc/articles/PMC5714504/) | Sex differentiation, part of primary gene list used at the UCLA… |
| JUN | Mouse | NA | https://academic.oup.com/biolreprod/article/102/6/1248/5811995 | Upregulated when female exposed to masculinizing substance |
| KAL1 | Human | NA | https://[www.ncbi.nlm.nih.gov/pmc/articles/PMC5714504/](http://www.ncbi.nlm.nih.gov/pmc/articles/PMC5714504/) | Hypogonadism, part of primary gene list used at the UCLA… |
| KDM5D | Human | ENSMMUG00000045017.1 | https://[www.ncbi.nlm.nih.gov/pmc/articles/PMC5714504/](http://www.ncbi.nlm.nih.gov/pmc/articles/PMC5714504/) | Sex differentiation, part of primary gene list used at the UCLA… |
| KIF14 | Mouse | ENSMMUG00000004266.3 | https://[www.ncbi.nlm.nih.gov/pmc/articles/PMC4856068/](http://www.ncbi.nlm.nih.gov/pmc/articles/PMC4856068/) | Genes associated with bone size/density and part of QTL's associated with baculum variation |
| KISS1R | Human | NA | https://[www.ncbi.nlm.nih.gov/pmc/articles/PMC5714504/](http://www.ncbi.nlm.nih.gov/pmc/articles/PMC5714504/) | Hypogonadism, part of primary gene list used at the UCLA… |
| LEF1 | Mouse | ENSMMUG00000023256.3 | https://academic.oup.com/biolreprod/article/102/6/1248/5811995 | Upregulated when female exposed to masculinizing substance |
| LEP | Human | ENSMMUG00000005322.3 | https://[www.ncbi.nlm.nih.gov/pmc/articles/PMC5714504/](http://www.ncbi.nlm.nih.gov/pmc/articles/PMC5714504/) | Hypogonadism, part of primary gene list used at the UCLA… |
| LEPR | Human | ENSMMUG00000003265.3 | https://[www.ncbi.nlm.nih.gov/pmc/articles/PMC5714504/](http://www.ncbi.nlm.nih.gov/pmc/articles/PMC5714504/) | Hypogonadism, part of primary gene list used at the UCLA… |
| LHCGR | Human | NA | https://[www.ncbi.nlm.nih.gov/pmc/articles/PMC5714504/](http://www.ncbi.nlm.nih.gov/pmc/articles/PMC5714504/) | Sex differentiation, part of primary gene list used at the UCLA… |
| LHX3 | Human | ENSMMUG00000004882.3 | https://[www.ncbi.nlm.nih.gov/pmc/articles/PMC5714504/](http://www.ncbi.nlm.nih.gov/pmc/articles/PMC5714504/) | Hypogonadism, part of primary gene list used at the UCLA… |
| LHX8 | Human | ENSMMUG00000005287.3 | https://[www.ncbi.nlm.nih.gov/pmc/articles/PMC5866176/](http://www.ncbi.nlm.nih.gov/pmc/articles/PMC5866176/) | associated with ovarian dysgenesis and premature ovarian failure |
| LHX9 | Human | ENSMMUG00000012259.3 | https://[www.ncbi.nlm.nih.gov/pmc/articles/PMC5866176/](http://www.ncbi.nlm.nih.gov/pmc/articles/PMC5866176/) |  |
| LRP5 | Mouse | NA | https://academic.oup.com/biolreprod/article/102/6/1248/5811995 | Upregulated when female exposed to masculinizing substance |
| LRP6 | Mouse | ENSMMUG00000023317.3 | https://academic.oup.com/biolreprod/article/102/6/1248/5811995 | Upregulated when female exposed to masculinizing substance |
| MAFB | Mouse | ENSMMUG00000004148.3 | https://[www.ncbi.nlm.nih.gov/pmc/articles/PMC4856068/](http://www.ncbi.nlm.nih.gov/pmc/articles/PMC4856068/) | Abnormal ossification in inner ear |
| MAMLD1 | Human | ENSMMUG00000022263.3 | https://[www.ncbi.nlm.nih.gov/pmc/articles/PMC5714504/](http://www.ncbi.nlm.nih.gov/pmc/articles/PMC5714504/) | Sex differentiation, part of primary gene list used at the UCLA… |
| MAP3K1 | Human | ENSMMUG00000012387.3 | https://[www.ncbi.nlm.nih.gov/pmc/articles/PMC5714504/](http://www.ncbi.nlm.nih.gov/pmc/articles/PMC5714504/) | Sex determination, part of primary gene list used at the UCLA Clinical Genome Center to call DSD variants |
| MCM8 | Human | ENSMMUG00000013740.3 | https://[www.ncbi.nlm.nih.gov/pmc/articles/PMC5866176/](http://www.ncbi.nlm.nih.gov/pmc/articles/PMC5866176/) | associated with ovarian dysgenesis and premature ovarian failure |
| MCM9 | Human | NA | https://[www.ncbi.nlm.nih.gov/pmc/articles/PMC5866176/](http://www.ncbi.nlm.nih.gov/pmc/articles/PMC5866176/) | associated with ovarian dysgenesis and premature ovarian failure |
| MGAT2 | Mouse | NA | https://[www.ncbi.nlm.nih.gov/pmc/articles/PMC4856068/](http://www.ncbi.nlm.nih.gov/pmc/articles/PMC4856068/) | Genes associated with bone size/density and part of QTL's associated with baculum variation |
| MKKS | Human | ENSMMUG00000031166.2 | https://[www.ncbi.nlm.nih.gov/pmc/articles/PMC5714504/](http://www.ncbi.nlm.nih.gov/pmc/articles/PMC5714504/) | Hypogonadism, part of primary gene list used at the UCLA… |
| MSX1 | Mouse | NA | https://dev.biologists.org/content/develop/127/11/2471.full.pdf | expressed in the distal urethral plate epithelium of the GT |
| NLK | Mouse | ENSMMUG00000018719.2 | https://academic.oup.com/biolreprod/article/102/6/1248/5811995 | Upregulated when female exposed to masculinizing substance |
| NOBOX | Human | ENSMMUG00000020609.3 | https://[www.ncbi.nlm.nih.gov/pmc/articles/PMC5866176/](http://www.ncbi.nlm.nih.gov/pmc/articles/PMC5866176/) | ovarian dysgenesis and premature ovarian failure |
| NR0B1 | Human | ENSMMUG00000012775.3 | https://[www.ncbi.nlm.nih.gov/pmc/articles/PMC5714504/](http://www.ncbi.nlm.nih.gov/pmc/articles/PMC5714504/) | Sex determination, part of primary gene list used at the UCLA Clinical Genome Center to call DSD variants |
| NR5A1 | Human | ENSMMUG00000014111.3 | https://[www.ncbi.nlm.nih.gov/pmc/articles/PMC5714504/](http://www.ncbi.nlm.nih.gov/pmc/articles/PMC5714504/) | Sex determination, part of primary gene list used at the UCLA Clinical Genome Center to call DSD variants |
| PCSK1 | Human | ENSMMUG00000005134.3 | https://[www.ncbi.nlm.nih.gov/pmc/articles/PMC5714504/](http://www.ncbi.nlm.nih.gov/pmc/articles/PMC5714504/) | Hypogonadism, part of primary gene list used at the UCLA… |
| PLGC1 | Mouse | NA | https://[www.ncbi.nlm.nih.gov/pmc/articles/PMC4856068/](http://www.ncbi.nlm.nih.gov/pmc/articles/PMC4856068/) | Abnormal ebryonic growth; Abnormal adult growth |
| PPP2R5D | Mouse | ENSMMUG00000004873.3 | https://academic.oup.com/biolreprod/article/102/6/1248/5811995 | Upregulated when female exposed to masculinizing substance |
| PROK2 | Human | ENSMMUG00000018494.3 | https://[www.ncbi.nlm.nih.gov/pmc/articles/PMC5714504/](http://www.ncbi.nlm.nih.gov/pmc/articles/PMC5714504/) | Hypogonadism, part of primary gene list used at the UCLA… |
| PROKR2 | Human | ENSMMUG00000003713.2 | https://[www.ncbi.nlm.nih.gov/pmc/articles/PMC5714504/](http://www.ncbi.nlm.nih.gov/pmc/articles/PMC5714504/) | Hypogonadism, part of primary gene list used at the UCLA… |
| PROP1 | Human | ENSMMUG00000039065.1 | https://[www.ncbi.nlm.nih.gov/pmc/articles/PMC5714504/](http://www.ncbi.nlm.nih.gov/pmc/articles/PMC5714504/) | Hypogonadism, part of primary gene list used at the UCLA… |
| PTPN11 | Human | ENSMMUG00000013253.3 | https://[www.ncbi.nlm.nih.gov/pmc/articles/PMC5714504/](http://www.ncbi.nlm.nih.gov/pmc/articles/PMC5714504/) | Hypogonadism, part of primary gene list used at the UCLA… |
| PTPRC | Mouse | ENSMMUG00000000009.3 | https://[www.ncbi.nlm.nih.gov/pmc/articles/PMC4856068/](http://www.ncbi.nlm.nih.gov/pmc/articles/PMC4856068/) | Genes associated with bone size/density and part of QTL's associated with baculum variation |
| RBL1 | Mouse | ENSMMUG00000014376.3 | https://[www.ncbi.nlm.nih.gov/pmc/articles/PMC4856068/](http://www.ncbi.nlm.nih.gov/pmc/articles/PMC4856068/) | Genes associated with bone size/density and part of QTL's associated with baculum variation |
| RHOJ | Mouse | ENSMMUG00000008203.2 | https://[www.ncbi.nlm.nih.gov/pmc/articles/PMC4856068/](http://www.ncbi.nlm.nih.gov/pmc/articles/PMC4856068/) | Abnormal embryonic growth |
| RHOU | Mouse | ENSMMUG00000002924.3 | https://academic.oup.com/biolreprod/article/102/6/1248/5811995 | Upregulated when female exposed to masculinizing substance |
| RSPO1 | Human | ENSMMUG00000008420.3 | https://[www.ncbi.nlm.nih.gov/pmc/articles/PMC5714504/](http://www.ncbi.nlm.nih.gov/pmc/articles/PMC5714504/) | Sex determination, part of primary gene list used at the UCLA Clinical Genome Center to call DSD variants |
| SEC23A | Mouse | ENSMMUG00000003184.3 | https://[www.ncbi.nlm.nih.gov/pmc/articles/PMC4856068/](http://www.ncbi.nlm.nih.gov/pmc/articles/PMC4856068/) | Chondrogenesis regulation |
| SIX1 | Human | NA | https://[www.ncbi.nlm.nih.gov/pmc/articles/PMC5866176/](http://www.ncbi.nlm.nih.gov/pmc/articles/PMC5866176/) | initial differentiation of the bipotential gonad |
| SIX4 | Human | NA | https://[www.ncbi.nlm.nih.gov/pmc/articles/PMC5866176/](http://www.ncbi.nlm.nih.gov/pmc/articles/PMC5866176/) | initial differentiation of the bipotential gonad |
| SOS1 | Human | ENSMMUG00000020575.3 | https://[www.ncbi.nlm.nih.gov/pmc/articles/PMC5714504/](http://www.ncbi.nlm.nih.gov/pmc/articles/PMC5714504/) | Hypogonadism, part of primary gene list used at the UCLA… |
| SOX10 | Human | NA | https://[www.ncbi.nlm.nih.gov/pmc/articles/PMC5866176/](http://www.ncbi.nlm.nih.gov/pmc/articles/PMC5866176/) | relevant for testicular differentiation |
| SOX13 | Human | ENSMMUG00000008460.3 | https://[www.ncbi.nlm.nih.gov/pmc/articles/PMC5866176/](http://www.ncbi.nlm.nih.gov/pmc/articles/PMC5866176/) | relevant for testicular differentiation |
| SOX3 | Human | ENSMMUG00000013179.3 | https://pubmed.ncbi.nlm.nih.gov/31523625/ | Hypospladia and kidney issues |
| SOX8 | Human | NA | https://[www.ncbi.nlm.nih.gov/pmc/articles/PMC5866176/](http://www.ncbi.nlm.nih.gov/pmc/articles/PMC5866176/) | associated with XY gonadal dysgenesis |
| SOX9 | Human | ENSMMUG00000002412.3 | https://[www.ncbi.nlm.nih.gov/pmc/articles/PMC5714504/](http://www.ncbi.nlm.nih.gov/pmc/articles/PMC5714504/) | Sex determination, part of primary gene list used at the UCLA Clinical Genome Center to call DSD variants |
| SRD5A2 | Human | NA | https://[www.ncbi.nlm.nih.gov/pmc/articles/PMC5714504/](http://www.ncbi.nlm.nih.gov/pmc/articles/PMC5714504/) | Sex differentiation, part of primary gene list used at the UCLA… |
| SRY | Human | ENSMMUG00000041853.1 | https://[www.ncbi.nlm.nih.gov/pmc/articles/PMC5714504/](http://www.ncbi.nlm.nih.gov/pmc/articles/PMC5714504/) | Sex determination, part of primary gene list used at the UCLA Clinical Genome Center to call DSD variants |
| STAG3 | Human | ENSMMUG00000018405.3 | https://[www.ncbi.nlm.nih.gov/pmc/articles/PMC5714504/](http://www.ncbi.nlm.nih.gov/pmc/articles/PMC5714504/) | Sex determination, part of primary gene list used at the UCLA Clinical Genome Center to call DSD variants |
| STAR | Human | NA | https://[www.ncbi.nlm.nih.gov/pmc/articles/PMC5714504/](http://www.ncbi.nlm.nih.gov/pmc/articles/PMC5714504/) | Sex differentiation, part of primary gene list used at the UCLA… |
| STRP1 | Mouse | NA | https://academic.oup.com/biolreprod/article/102/6/1248/5811995 | Downregulated when females exposed to masculinizing substance |
| TAC3 | Human | ENSMMUG00000003203.3 | https://[www.ncbi.nlm.nih.gov/pmc/articles/PMC5714504/](http://www.ncbi.nlm.nih.gov/pmc/articles/PMC5714504/) | Hypogonadism, part of primary gene list used at the UCLA… |
| TACR3 | Human | NA | https://[www.ncbi.nlm.nih.gov/pmc/articles/PMC5714504/](http://www.ncbi.nlm.nih.gov/pmc/articles/PMC5714504/) | Hypogonadism, part of primary gene list used at the UCLA… |
| TBX3 | Human | ENSMMUG00000015070.3 | https://[www.nature.com/articles/ng0797-311](http://www.nature.com/articles/ng0797-311) | Mutation associated with a disorder which includes abnormal genital development |
| TCF7L1 | Mouse | ENSMMUG00000020134.3 | https://academic.oup.com/biolreprod/article/102/6/1248/5811995 | Upregulated when female exposed to masculinizing substance |
| TGM2 | Mouse | ENSMMUG00000018925.3 | https://[www.ncbi.nlm.nih.gov/pmc/articles/PMC4856068/](http://www.ncbi.nlm.nih.gov/pmc/articles/PMC4856068/) | Genes associated with bone size/density and part of QTL's associated with baculum variation |
| TRIM32 | Human | ENSMMUG00000013107.3 | https://[www.ncbi.nlm.nih.gov/pmc/articles/PMC5714504/](http://www.ncbi.nlm.nih.gov/pmc/articles/PMC5714504/) | Hypogonadism, part of primary gene list used at the UCLA… |
| TTC8 | Human | ENSMMUG00000004612.3 | https://[www.ncbi.nlm.nih.gov/pmc/articles/PMC5714504/](http://www.ncbi.nlm.nih.gov/pmc/articles/PMC5714504/) | Hypogonadism, part of primary gene list used at the UCLA… |
| VAMP7 | Human | ENSMMUG00000002015.3 | https://[www.ncbi.nlm.nih.gov/pmc/articles/PMC5714504/](http://www.ncbi.nlm.nih.gov/pmc/articles/PMC5714504/) | Sex differentiation, part of primary gene list used at the UCLA… |
| WISP1 | Mouse | NA | https://academic.oup.com/biolreprod/article/102/6/1248/5811995 | Downregulated when females exposed to masculinizing substance |
| WNT2 | Mouse | ENSMMUG00000047811.1 | https://academic.oup.com/biolreprod/article/102/6/1248/5811995 | Upregulated when female exposed to masculinizing substance |
| WNT4 | Human | NA | https://[www.ncbi.nlm.nih.gov/pmc/articles/PMC5714504/](http://www.ncbi.nlm.nih.gov/pmc/articles/PMC5714504/) | Sex determination, part of primary gene list used at the UCLA Clinical Genome Center to call DSD variants |
| WNT5A | Mouse | ENSMMUG00000039130.1 | https://academic.oup.com/biolreprod/article/102/6/1248/5811995 | Upregulated when female exposed to masculinizing substance |
| WT1 | Human | ENSMMUG00000009881.3 | https://pubmed.ncbi.nlm.nih.gov/32780953/; https://pubmed.ncbi.nlm.nih.gov/32493750/ | mutation associated with XY gonadal dysgenesis; increase occurrence of variance in those with specific DSD |
| WWOX | Human | ENSMMUG00000010610.3 | https://[www.ncbi.nlm.nih.gov/pmc/articles/PMC5714504/](http://www.ncbi.nlm.nih.gov/pmc/articles/PMC5714504/) | Sex determination, part of primary gene list used at the UCLA Clinical Genome Center to call DSD variants |
| ZFPM2 | Human | ENSMMUG00000007721.3 | https://[www.ncbi.nlm.nih.gov/pmc/articles/PMC5714504/](http://www.ncbi.nlm.nih.gov/pmc/articles/PMC5714504/) | Sex determination, part of primary gene list used at the UCLA Clinical Genome Center to call DSD variants |

**Table S5.** Permutation results for genes unqie to male genital morphology as compared to female genital morphology.

| **Gene set** | **Number of genes** | **dN/dS Arctoides** | **Fst Arc-Sin** | **Fst Arc-Fas** | **Dxy Arc-Sin** | **Dxy Arc-Fas** | **Fdm pi** |
| --- | --- | --- | --- | --- | --- | --- | --- |
| Male genes | 1192 (347 dN/dS) | 0.141 | 0.369 | 0.476 | 0.147 | 0.199 | -0.166 0.050 |
| Female genes | 444 (115 dN/dS) | 0.101 | 0.375 | 0.472 | 0.150 | 0.196 | -0.151 0.048 |
| Permutation test p-value |  | 0.240 | 0.460 | 0.440 | 0.120 | 0.120 | 0.020 0.180 |

**Table S6.** List of population genetic tests for outliers, the total number of genes analyzed, the number of Candidate Genes (CGs) along with the resulting number of total outliers and CG outliers. A chi-square test was done to compare the proportion of expected outliers that were not candidate genes to the observed percentage of candidate gene outliers. Further, for each metric, the mean of all genes is compared against the CGs in a permutation test with the results shown here.

| **Metric** | **Number of genes** | **Number CGs** | **Correlation with RR** | **Total # outliers** | **CG outliers** | **Expected outlier %** | **Observed outlier %** | **p-value** | **Mean ALL genes** | **Mean CGs** | **Permutation test** |
| --- | --- | --- | --- | --- | --- | --- | --- | --- | --- | --- | --- |
| dN/dS Arctoides | 1903 | 313 | n/a | 97 | 21 | 4.78% | 6.71% | 0.230 | 0.143 | 0.160 | 0.7 |
| Fst Arc-Sin | 10543 | 1055 | < 2.2e-16 | 139 | 12 | 1.34% | 1.14% | 0.694 | 0.339 | 0.365 | **0** |
| Fst Arc-Fas | 10544 | 1055 | < 2.2e-16 | 94 | 8 | 0.91% | 0.76% | 0.758 | 0.452 | 0.472 | **0** |
| Dxy Arc-Sin | 10543 | 1055 | < 2.2e-16 | 204 | 10 | 2.04% | 0.95% | **0.022** | 0.148 | 0.148 | 0.3 |
| Dxy Arc-Fas | 10544 | 1055 | 4.28E-16 | 176 | 16 | 1.69% | 1.52% | 0.784 | 0.194 | 0.198 | **0** |
| Fdm upper | 9181 | 1037 | < 2.2e-16 | 87 | 7 | 0.98% | 0.09% | 0.433 | -0.161 | -0.164 | 0.38 |
| Fdm lower |  |  |  | 155 | 13 | 1.74% | 0.14% | 0.313 |  |  |  |
| pi | 10546 | 1055 | < 2.2e-16 | 91 | 6 | 0.90% | 0.57% | 0.365 | 0.055 | 0.051 | **0** |
| TOTAL |  |  |  |  | 67 |  |  |  |  |  |  |

**Table S7.** Outlier Candidate Genes across seven population genetic metrics. For each gene, the ensGene ID and GeneID are provided along with the metric of each population genetic measure used to identify outliers. Additionally, the average recombination rate of the gene is also included.

| **ensembl Gene ID** | **ensembl gene name** | **MP outlier** | **HPO outlier** | **pi in *M. arctoides*** | **fdM** | **RecRate** | **Arc.dNdS** | **Fst_Arctoides_Fascicularis** | **dxy_Arctoides_Fascicularis** | **Fst_Arctoides_Sinica** | **dxy_Arctoides_Sinica** | **pi_outlier** | **dnds_outlier** | **fdm_upper_outlier** | **fdm_lower_outlier** |
| --- | --- | --- | --- | --- | --- | --- | --- | --- | --- | --- | --- | --- | --- | --- | --- |
| ENSMMUG00000017977.3 | ADAMTS3 | 0 | 1 | 0.0689 | -0.1578 | 0.50995 | 1.27209 | 0.4461 | 0.194 | 0.3093 | 0.1477 | 0 | 1 | 0 | 0 |
| ENSMMUG00000004168.3 | AIRE | 0 | 1 | 0.0115 | -0.4963 | 1.36513 | NA | 0.759 | 0.3336 | 0.4546 | 0.139 | 0 | 0 | 0 | 1 |
| **ENSMMUG00000000245.3** | **ASPM** | **1** | **1** | **0.0504** | **0.001** | **0.184588** | **0.73929** | **0.2525** | **0.1449** | **0.2376** | **0.1543** | **0** | **1** | **0** | **0** |
| ENSMMUG00000007180.3 | BTBD8 | 1 | 0 | 0.0298 | -0.0419 | 0.222272 | 1.12428 | 0.5938 | 0.207 | 0.6234 | 0.2292 | 0 | 1 | 0 | 0 |
| ENSMMUG00000016480.3 | CAMKV | 1 | 0 | 0.0115 | -0.1333 | 0.0332222 | NA | 0.7122 | 0.3558 | 0.7674 | 0.2075 | 0 | 0 | 0 | 0 |
| ENSMMUG00000022962.3 | CCR8 | 1 | 0 | 0.0143 | -0.3809 | 0.173478 | NA | 0.7609 | 0.3744 | 0.7133 | 0.1776 | 0 | 0 | 0 | 0 |
| ENSMMUG00000000688.3 | CD96 | 0 | 1 | 0.0772 | -0.222 | 0.378614 | 0.8514 | 0.4481 | 0.2011 | 0.268 | 0.1789 | 0 | 1 | 0 | 0 |
| ENSMMUG00000003773.3 | CEP19 | 0 | 1 | 0.0293 | -0.0615 | 2.98048 | NA | 0.6488 | 0.281 | 0.5216 | 0.1669 | 1 | 0 | 0 | 0 |
| ENSMMUG00000013448.3 | CFAP44 | 1 | 0 | 0.0478 | -0.5378 | 0.224046 | NA | 0.6409 | 0.2783 | 0.229 | 0.0948 | 0 | 0 | 0 | 1 |
| ENSMMUG00000022166.3 | CPLANE1 | 0 | 1 | 0.0103 | -0.2237 | 0.176621 | 0.887914 | 0.6635 | 0.2346 | 0.5059 | 0.1423 | 0 | 1 | 0 | 0 |
| ENSMMUG00000014423.3 | CSPP1 | 0 | 1 | 0.0151 | -0.051 | 0.101747 | 1.08172 | 0.806 | 0.2689 | 0.8063 | 0.2668 | 0 | 1 | 0 | 0 |
| **ENSMMUG00000017601.3** | **CYP17A1** | **1** | **1** | **0.044** | **-0.5671** | **0.223304** | **NA** | **0.6396** | **0.2755** | **0.3516** | **0.1002** | **0** | **0** | **0** | **1** |
| **ENSMMUG00000000755.3** | **CYP21A2** | **0** | **1** | **0.1751** | **-0.0294** | **2.20096** | **NA** | **0.1753** | **0.2248** | **0.0685** | **0.2103** | **0** | **0** | **0** | **0** |
| **ENSMMUG00000001090.3** | **DACT1** | **1** | **1** | **0.0429** | **0.1491** | **0.254594** | **1.00938** | **0.3702** | **0.154** | **0.4451** | **0.1906** | **0** | **1** | **0** | **0** |
| ENSMMUG00000004480.3 | DND1 | 1 | 0 | 0.0415 | -0.2134 | 0.0127778 | NA | 0.8176 | 0.3382 | 0.5475 | 0.2403 | 0 | 0 | 0 | 0 |
| ENSMMUG00000009865.3 | DPY19L2 | 1 | 0 | 0.3103 | -0.1687 | 0.536707 | NA | 0.0651 | 0.3044 | -0.0333 | 0.2796 | 0 | 0 | 0 | 0 |
| ENSMMUG00000008761.3 | EXO1 | 1 | 0 | 0.0079 | 0.0112 | 0.883867 | 0.666824 | 0.508 | 0.1884 | 0.4947 | 0.1862 | 0 | 1 | 0 | 0 |
| ENSMMUG00000041338.1 | EYS | 0 | 1 | 0.0751 | -0.1384 | 0.610325 | 1.05867 | 0.3508 | 0.1731 | 0.2427 | 0.1493 | 0 | 1 | 0 | 0 |
| ENSMMUG00000011186.3 | FAS | 1 | 1 | 0.0929 | 0.2347 | 0.376017 | NA | 0.2842 | 0.1559 | 0.3842 | 0.2073 | 0 | 0 | 1 | 0 |
| **ENSMMUG00000006944.3** | **GATA3** | **0** | **1** | **0.0395** | **-0.1861** | **4.30505** | **NA** | **0.5312** | **0.2** | **0.3381** | **0.1354** | **1** | **0** | **0** | **1** |
| ENSMMUG00000019998.3 | GFM2 | 0 | 1 | 0.0285 | -0.5448 | 0.263932 | NA | 0.7445 | 0.2991 | 0.2366 | 0.0579 | 0 | 0 | 0 | 1 |
| **ENSMMUG00000023733.3** | **HFE** | **0** | **1** | **0.0158** | **0.1058** | **0.0566579** | **NA** | **0.453** | **0.1787** | **0.7287** | **0.2704** | **0** | **0** | **0** | **0** |
| ENSMMUG00000006819.3 | HFM1 | 1 | 1 | 0.0966 | 0.1271 | 0.164793 | 0.913823 | 0.3735 | 0.1907 | 0.3677 | 0.2154 | 0 | 1 | 0 | 0 |
| ENSMMUG00000019529.3 | HMGA1 | 1 | 0 | 0.0424 | -0.0983 | 2.95922 | NA | 0.529 | 0.205 | 0.3846 | 0.1587 | 1 | 0 | 0 | 0 |
| ENSMMUG00000009396.3 | HSPA4 | 1 | 0 | 0.035 | -0.0113 | 0.114299 | 1.00E-04 | 0.6261 | 0.2259 | 0.7813 | 0.2463 | 0 | 0 | 0 | 0 |
| ENSMMUG00000023569.3 | IL10 | 0 | 1 | 0.0563 | -0.6205 | 0.0995667 | NA | 0.6475 | 0.2331 | 0.1464 | 0.0634 | 0 | 0 | 0 | 1 |
| ENSMMUG00000023277.3 | INPP5B | 1 | 0 | 0.033 | -0.3848 | 0.842782 | 1.06559 | 0.5391 | 0.2228 | 0.3149 | 0.1093 | 0 | 1 | 0 | 0 |
| ENSMMUG00000000455.3 | KDM3B | 0 | 1 | 0.0236 | 0.2238 | 0.0567772 | NA | 0.4772 | 0.157 | 0.7598 | 0.2524 | 0 | 0 | 1 | 0 |
| ENSMMUG00000001084.3 | KIAA0586 | 0 | 1 | 0.0566 | -0.1847 | 0.354904 | 0.665304 | 0.5164 | 0.2027 | 0.3739 | 0.1399 | 0 | 1 | 0 | 0 |
| ENSMMUG00000039441.1 | MAB21L2 | 0 | 1 | 0.0336 | -0.2468 | 0.17014 | NA | 0.6351 | 0.3573 | 0.7036 | 0.1698 | 0 | 0 | 0 | 0 |
| ENSMMUG00000005298.3 | MAN2B2 | 1 | 0 | 0.0655 | -0.0047 | 0.858279 | 0.680223 | 0.4519 | 0.1774 | 0.3507 | 0.179 | 0 | 1 | 0 | 0 |
| ENSMMUG00000003739.3 | MANBA | 1 | 0 | 0.0319 | -0.516 | 0.976342 | 0.402567 | 0.5779 | 0.2477 | 0.2389 | 0.114 | 0 | 0 | 0 | 1 |
| ENSMMUG00000004766.3 | MINPP1 | 0 | 1 | 0.0535 | -0.6286 | 0.188468 | 1.00E-04 | 0.4964 | 0.2326 | 0.1359 | 0.0901 | 0 | 0 | 0 | 1 |
| ENSMMUG00000003353.3 | MST1R | 1 | 0 | 0.0088 | -0.1602 | 0.0718519 | NA | 0.7036 | 0.2839 | 0.7797 | 0.2067 | 0 | 0 | 0 | 0 |
| ENSMMUG00000014818.3 | MYLK | 0 | 1 | 0.0706 | -0.2908 | 0.597102 | 3.17448 | 0.4588 | 0.2168 | 0.2559 | 0.1489 | 0 | 1 | 0 | 0 |
| ENSMMUG00000023171.3 | NODAL | 0 | 1 | 0.0198 | -0.3838 | 0.171792 | NA | 0.7273 | 0.3491 | 0.3895 | 0.1648 | 0 | 0 | 0 | 0 |
| ENSMMUG00000022888.3 | NPHP4 | 0 | 1 | 0.0604 | -0.1281 | 0.630321 | 0.679937 | 0.3851 | 0.1718 | 0.2823 | 0.1384 | 0 | 1 | 0 | 0 |
| ENSMMUG00000005430.3 | NR2F2 | 1 | 1 | 0.0263 | -0.2644 | 2.2393 | NA | 0.7294 | 0.2194 | 0.3883 | 0.1119 | 0 | 0 | 0 | 0 |
| ENSMMUG00000012914.3 | PCNT | 0 | 1 | 0.0491 | -0.1732 | 0.237539 | 1.6082 | 0.4471 | 0.2052 | 0.3124 | 0.1297 | 0 | 1 | 0 | 0 |
| ENSMMUG00000007228.3 | PGBD1 | 1 | 0 | 0.0337 | 0.2154 | 0.151377 | 0.240662 | 0.2813 | 0.1059 | 0.2589 | 0.1423 | 0 | 0 | 1 | 0 |
| ENSMMUG00000005188.3 | PIK3CA | 1 | 1 | 0.0465 | -0.5781 | 0.13414 | NA | 0.7607 | 0.3312 | 0.4181 | 0.1025 | 0 | 0 | 0 | 1 |
| ENSMMUG00000016244.2 | POU1F1 | 0 | 1 | 0.0302 | 0.2785 | 0.650553 | NA | 0.2601 | 0.1185 | 0.5988 | 0.2097 | 0 | 0 | 1 | 0 |
| ENSMMUG00000016301.3 | PRPF3 | 0 | 1 | 0.0323 | -0.4561 | 0.018 | NA | 0.7768 | 0.3294 | 0.5278 | 0.1254 | 0 | 0 | 0 | 0 |
| ENSMMUG00000011301.3 | PSMB8 | 0 | 1 | 0.2278 | -0.1452 | 2.85454 | NA | 0.0571 | 0.2289 | 0.0383 | 0.241 | 0 | 0 | 0 | 0 |
| ENSMMUG00000014045.3 | PTPN23 | 0 | 1 | 0.0083 | -0.2205 | 3.08924 | 1.00E-04 | 0.6252 | 0.2342 | 0.4134 | 0.1329 | 1 | 0 | 0 | 0 |
| **ENSMMUG00000000009.3** | **PTPRC** | **0** | **1** | **0.0805** | **-0.3009** | **0.65054** | **1.26709** | **0.5386** | **0.2427** | **0.2511** | **0.1609** | **0** | **1** | **0** | **0** |
| ENSMMUG00000007450.3 | RAF1 | 0 | 1 | 0.0631 | 0.2121 | 0.194951 | NA | 0.2463 | 0.1137 | 0.5415 | 0.2221 | 0 | 0 | 1 | 0 |
| ENSMMUG00000049179.1 | RF00108 | 0 | 1 | 0.0134 | 0.2747 | 0.0622174 | NA | 0.3296 | 0.144 | 0.6462 | 0.2377 | 0 | 0 | 1 | 0 |
| ENSMMUG00000040774.1 | RNF148 | 1 | 0 | 0.0513 | -0.5897 | 0.00472727 | NA | 0.5725 | 0.2666 | 0.196 | 0.0884 | 0 | 0 | 0 | 1 |
| ENSMMUG00000043605.1 | RP1L1 | 0 | 1 | 0.051 | -0.3429 | 0.602917 | 1.89268 | 0.4129 | 0.2061 | 0.3181 | 0.1399 | 0 | 1 | 0 | 0 |
| ENSMMUG00000019994.3 | SDCCAG8 | 0 | 1 | 0.0253 | -0.1529 | 0.344475 | 0.800398 | 0.6057 | 0.2179 | 0.4937 | 0.1585 | 0 | 1 | 0 | 0 |
| ENSMMUG00000009281.3 | SIN3A | 0 | 1 | 0.0358 | -0.5621 | 0.140179 | NA | 0.7949 | 0.402 | 0.4783 | 0.1456 | 0 | 0 | 0 | 1 |
| ENSMMUG00000023647.3 | SKIDA1 | 1 | 0 | 0.0367 | -0.034 | 0.0462308 | NA | 0.6379 | 0.2868 | 0.7838 | 0.2357 | 0 | 0 | 0 | 0 |
| ENSMMUG00000000645.2 | SLC35D1 | 0 | 1 | 0.0138 | -0.0254 | 0.114636 | 1.00E-04 | 0.5945 | 0.2026 | 0.7811 | 0.2261 | 0 | 0 | 0 | 0 |
| ENSMMUG00000003789.3 | SLC9A3 | 1 | 0 | 0.0146 | -0.1404 | 1.95234 | NA | 0.5062 | 0.1973 | 0.4402 | 0.1363 | 1 | 0 | 0 | 0 |
| ENSMMUG00000016195.3 | SNAI2 | 1 | 0 | 0.0581 | -0.3413 | 5.66727 | NA | 0.3915 | 0.1795 | 0.1499 | 0.0887 | 1 | 0 | 0 | 1 |
| ENSMMUG00000003263.3 | STAG1 | 1 | 1 | 0.0244 | -0.4082 | 0.0797506 | NA | 0.7854 | 0.3285 | 0.6865 | 0.157 | 0 | 0 | 0 | 0 |
| ENSMMUG00000022763.2 | TACSTD2 | 1 | 0 | 0.0553 | 0.0269 | 0.199254 | NA | 0.6455 | 0.2988 | 0.5916 | 0.3176 | 0 | 0 | 0 | 0 |
| ENSMMUG00000009096.2 | TDRKH | 1 | 0 | 0.0705 | -0.1028 | 2.0054 | NA | 0.633 | 0.2606 | 0.2973 | 0.1795 | 0 | 0 | 0 | 0 |
| ENSMMUG00000006186.3 | TFAP2A | 0 | 1 | 0.032 | -0.0277 | 1.68915 | NA | 0.5184 | 0.2029 | 0.6038 | 0.1828 | 0 | 0 | 0 | 0 |
| ENSMMUG00000015847.3 | TLR2 | 0 | 1 | 0.0408 | -0.1112 | 0.28334 | 0.956263 | 0.5727 | 0.1951 | 0.2247 | 0.1628 | 0 | 1 | 0 | 0 |
| ENSMMUG00000015168.3 | TMED10 | 1 | 0 | 0.019 | -0.605 | 0.203899 | NA | 0.8461 | 0.4019 | 0.6361 | 0.137 | 0 | 0 | 0 | 1 |
| ENSMMUG00000010860.3 | TOGARAM1 | 0 | 1 | 0.0108 | -0.3366 | 0.103629 | 1.00E-04 | 0.795 | 0.3508 | 0.7577 | 0.1627 | 0 | 0 | 0 | 0 |
| ENSMMUG00000015127.3 | TOPAZ1 | 1 | 0 | 0.0565 | -0.2712 | 0.0598433 | 4.11511 | 0.4332 | 0.1701 | 0.2503 | 0.0995 | 0 | 1 | 0 | 0 |
| ENSMMUG00000020666.3 | UBE2D2 | 1 | 0 | 0.0092 | -0.123 | 1.4828 | NA | 0.6964 | 0.2268 | 0.6911 | 0.1436 | 0 | 0 | 0 | 0 |
| ENSMMUG00000020138.3 | WDR48 | 1 | 0 | 0.0652 | 0.1855 | 0.0280845 | NA | 0.3466 | 0.1367 | 0.4838 | 0.2143 | 0 | 0 | 1 | 0 |
| ENSMMUG00000012720.3 | ZCWPW1 | 1 | 0 | 0.0193 | -0.0193 | 0.147829 | NA | 0.6665 | 0.1996 | 0.787 | 0.2729 | 0 | 0 | 0 | 0 |

| **ensembl gene name** | **fst_arc_sin_outlier** | **fst_arc_fas_outlier** | **dxy_arc_sin_outlier** | **dxy_arc_fas_outlier** | **Multiple Outliers** | **HPO811_list** | **HPO10461_list** | **HPO10460_list** | **HPO812_list** | **HPO1827_list** | **HPO12244_list** | **HPO3252_list** | **Lit_CG_list** | **MP_0002210_list** | **MP_0003936_list** | **MP_0009198_list** |
| --- | --- | --- | --- | --- | --- | --- | --- | --- | --- | --- | --- | --- | --- | --- | --- | --- |
| ADAMTS3 | 0 | 0 | 0 | 0 | 1 | 1 | 1 | 0 | 0 | 0 | 0 | 0 | 0 | 0 | 0 | 0 |
| AIRE | 0 | 1 | 0 | 1 | 3 | 0 | 0 | 1 | 1 | 0 | 0 | 0 | 0 | 0 | 0 | 0 |
| **ASPM** | **0** | **0** | **0** | **0** | **1** | **0** | **0** | **0** | **0** | **0** | **0** | **0** | **1** | **1** | **0** | **1** |
| BTBD8 | 0 | 0 | 0 | 0 | 1 | 0 | 0 | 0 | 0 | 0 | 0 | 0 | 0 | 0 | 0 | 1 |
| CAMKV | 1 | 0 | 0 | 1 | 2 | 0 | 0 | 0 | 0 | 0 | 0 | 0 | 0 | 0 | 0 | 0 |
| CCR8 | 0 | 0 | 0 | 1 | 1 | 0 | 0 | 0 | 0 | 0 | 0 | 0 | 0 | 0 | 0 | 0 |
| CD96 | 0 | 0 | 0 | 0 | 1 | 1 | 1 | 1 | 0 | 0 | 0 | 0 | 0 | 0 | 0 | 0 |
| CEP19 | 1 | 1 | 0 | 1 | 4 | 1 | 1 | 1 | 1 | 0 | 0 | 0 | 0 | 0 | 0 | 0 |
| CFAP44 | 0 | 0 | 0 | 0 | 1 | 0 | 0 | 0 | 0 | 0 | 0 | 0 | 0 | 0 | 0 | 1 |
| CPLANE1 | 0 | 0 | 0 | 0 | 1 | 1 | 1 | 0 | 0 | 0 | 0 | 0 | 0 | 0 | 0 | 0 |
| CSPP1 | 1 | 0 | 1 | 0 | 3 | 1 | 1 | 0 | 0 | 0 | 0 | 0 | 0 | 0 | 0 | 0 |
| **CYP17A1** | **0** | **0** | **0** | **0** | **1** | **1** | **1** | **1** | **1** | **0** | **1** | **0** | **1** | **0** | **0** | **1** |
| **CYP21A2** | **0** | **0** | **1** | **0** | **1** | **1** | **1** | **0** | **0** | **0** | **0** | **0** | **1** | **0** | **0** | **0** |
| **DACT1** | **0** | **0** | **0** | **0** | **1** | **1** | **1** | **1** | **1** | **0** | **0** | **0** | **1** | **0** | **1** | **1** |
| DND1 | 0 | 0 | 1 | 1 | 2 | 0 | 0 | 0 | 0 | 0 | 0 | 0 | 0 | 1 | 0 | 1 |
| DPY19L2 | 0 | 0 | 1 | 0 | 1 | 0 | 0 | 0 | 0 | 0 | 0 | 0 | 0 | 1 | 0 | 1 |
| EXO1 | 0 | 0 | 0 | 0 | 1 | 0 | 0 | 0 | 0 | 0 | 0 | 0 | 0 | 1 | 0 | 1 |
| EYS | 0 | 0 | 0 | 0 | 1 | 1 | 1 | 0 | 0 | 0 | 0 | 0 | 0 | 0 | 0 | 0 |
| FAS | 0 | 0 | 0 | 0 | 1 | 1 | 1 | 1 | 1 | 0 | 0 | 0 | 0 | 0 | 0 | 0 |
| **GATA3** | **1** | **1** | **0** | **0** | **4** | **0** | **0** | **1** | **1** | **1** | **0** | **0** | **1** | **0** | **0** | **0** |
| GFM2 | 0 | 0 | 0 | 0 | 1 | 1 | 1 | 0 | 0 | 0 | 0 | 0 | 0 | 0 | 0 | 0 |
| **HFE** | **0** | **0** | **1** | **0** | **1** | **1** | **1** | **0** | **0** | **0** | **0** | **0** | **1** | **0** | **0** | **0** |
| HFM1 | 0 | 0 | 0 | 0 | 1 | 0 | 0 | 1 | 1 | 0 | 0 | 0 | 0 | 1 | 0 | 1 |
| HMGA1 | 0 | 1 | 0 | 0 | 2 | 0 | 0 | 0 | 0 | 0 | 0 | 0 | 0 | 1 | 0 | 1 |
| HSPA4 | 1 | 0 | 1 | 0 | 2 | 0 | 0 | 0 | 0 | 0 | 0 | 0 | 0 | 1 | 0 | 1 |
| IL10 | 0 | 0 | 0 | 0 | 1 | 1 | 1 | 0 | 0 | 0 | 0 | 0 | 0 | 0 | 0 | 0 |
| INPP5B | 0 | 0 | 0 | 0 | 1 | 0 | 0 | 0 | 0 | 0 | 0 | 0 | 0 | 0 | 0 | 1 |
| KDM3B | 0 | 0 | 1 | 0 | 2 | 1 | 1 | 0 | 0 | 0 | 0 | 0 | 0 | 0 | 0 | 0 |
| KIAA0586 | 0 | 0 | 0 | 0 | 1 | 1 | 1 | 0 | 0 | 0 | 0 | 0 | 0 | 0 | 0 | 0 |
| MAB21L2 | 0 | 0 | 0 | 1 | 1 | 1 | 1 | 0 | 0 | 0 | 0 | 0 | 0 | 0 | 0 | 0 |
| MAN2B2 | 0 | 0 | 0 | 0 | 1 | 0 | 0 | 0 | 0 | 0 | 0 | 0 | 0 | 0 | 0 | 0 |
| MANBA | 0 | 0 | 0 | 0 | 1 | 0 | 0 | 0 | 0 | 0 | 0 | 0 | 0 | 0 | 0 | 1 |
| MINPP1 | 0 | 0 | 0 | 0 | 1 | 1 | 1 | 1 | 1 | 0 | 0 | 0 | 0 | 0 | 0 | 0 |
| MST1R | 1 | 0 | 0 | 0 | 1 | 0 | 0 | 0 | 0 | 0 | 0 | 0 | 0 | 1 | 0 | 0 |
| MYLK | 0 | 0 | 0 | 0 | 1 | 1 | 1 | 0 | 0 | 0 | 0 | 0 | 0 | 0 | 0 | 0 |
| NODAL | 0 | 0 | 0 | 1 | 1 | 1 | 1 | 0 | 0 | 0 | 0 | 0 | 0 | 0 | 0 | 0 |
| NPHP4 | 0 | 0 | 0 | 0 | 1 | 0 | 0 | 1 | 1 | 0 | 0 | 0 | 0 | 0 | 0 | 0 |
| NR2F2 | 0 | 1 | 0 | 0 | 1 | 1 | 1 | 1 | 1 | 0 | 0 | 0 | 0 | 1 | 0 | 0 |
| PCNT | 0 | 0 | 0 | 0 | 1 | 1 | 1 | 1 | 0 | 0 | 0 | 0 | 0 | 0 | 0 | 0 |
| PGBD1 | 0 | 0 | 0 | 0 | 1 | 0 | 0 | 0 | 0 | 0 | 0 | 0 | 0 | 1 | 0 | 1 |
| PIK3CA | 0 | 0 | 0 | 1 | 2 | 1 | 1 | 1 | 1 | 0 | 0 | 0 | 0 | 0 | 0 | 0 |
| POU1F1 | 0 | 0 | 0 | 0 | 1 | 1 | 1 | 0 | 0 | 0 | 0 | 0 | 0 | 0 | 0 | 0 |
| PRPF3 | 0 | 0 | 0 | 1 | 1 | 1 | 1 | 0 | 0 | 0 | 0 | 0 | 0 | 0 | 0 | 0 |
| PSMB8 | 0 | 0 | 1 | 1 | 2 | 0 | 1 | 0 | 1 | 0 | 0 | 0 | 0 | 0 | 0 | 0 |
| PTPN23 | 1 | 1 | 0 | 1 | 4 | 1 | 1 | 0 | 0 | 0 | 0 | 0 | 0 | 0 | 0 | 0 |
| **PTPRC** | **0** | **0** | **0** | **0** | **1** | **0** | **0** | **0** | **0** | **0** | **0** | **0** | **1** | **0** | **0** | **0** |
| RAF1 | 0 | 0 | 0 | 0 | 1 | 1 | 1 | 0 | 0 | 0 | 0 | 0 | 0 | 0 | 0 | 0 |
| RF00108 | 0 | 0 | 0 | 0 | 1 | 1 | 1 | 1 | 0 | 0 | 0 | 0 | 0 | 0 | 0 | 0 |
| RNF148 | 0 | 0 | 0 | 0 | 1 | 0 | 0 | 0 | 0 | 0 | 0 | 0 | 0 | 0 | 0 | 1 |
| RP1L1 | 0 | 0 | 0 | 0 | 1 | 1 | 1 | 0 | 0 | 0 | 0 | 0 | 0 | 0 | 0 | 0 |
| SDCCAG8 | 0 | 0 | 0 | 0 | 1 | 1 | 1 | 1 | 1 | 0 | 0 | 0 | 0 | 0 | 0 | 0 |
| SIN3A | 0 | 0 | 0 | 1 | 2 | 1 | 1 | 0 | 0 | 0 | 0 | 0 | 0 | 0 | 0 | 0 |
| SKIDA1 | 1 | 0 | 0 | 0 | 1 | 0 | 0 | 0 | 0 | 0 | 0 | 0 | 0 | 0 | 0 | 0 |
| SLC35D1 | 1 | 0 | 0 | 0 | 1 | 1 | 1 | 0 | 0 | 0 | 0 | 0 | 0 | 0 | 0 | 0 |
| SLC9A3 | 0 | 0 | 0 | 0 | 1 | 0 | 0 | 0 | 0 | 0 | 0 | 0 | 0 | 0 | 0 | 1 |
| SNAI2 | 0 | 1 | 0 | 0 | 3 | 0 | 0 | 0 | 0 | 0 | 0 | 0 | 0 | 1 | 0 | 1 |
| STAG1 | 0 | 0 | 0 | 1 | 1 | 1 | 1 | 0 | 0 | 0 | 0 | 0 | 0 | 0 | 0 | 0 |
| TACSTD2 | 0 | 0 | 1 | 0 | 1 | 0 | 0 | 0 | 0 | 0 | 0 | 0 | 0 | 1 | 0 | 1 |
| TDRKH | 0 | 1 | 0 | 1 | 2 | 0 | 0 | 0 | 0 | 0 | 0 | 0 | 0 | 1 | 0 | 1 |
| TFAP2A | 1 | 0 | 0 | 0 | 1 | 1 | 1 | 0 | 0 | 0 | 0 | 0 | 0 | 0 | 0 | 0 |
| TLR2 | 0 | 0 | 0 | 0 | 1 | 0 | 0 | 1 | 1 | 0 | 0 | 0 | 0 | 0 | 0 | 0 |
| TMED10 | 0 | 0 | 0 | 1 | 2 | 0 | 0 | 0 | 0 | 0 | 0 | 0 | 0 | 1 | 0 | 1 |
| TOGARAM1 | 0 | 0 | 0 | 1 | 1 | 1 | 1 | 0 | 0 | 0 | 0 | 0 | 0 | 0 | 0 | 0 |
| TOPAZ1 | 0 | 0 | 0 | 0 | 1 | 0 | 0 | 0 | 0 | 0 | 0 | 0 | 0 | 1 | 0 | 1 |
| UBE2D2 | 1 | 0 | 0 | 0 | 1 | 0 | 0 | 0 | 0 | 0 | 0 | 0 | 0 | 1 | 0 | 0 |
| WDR48 | 0 | 0 | 0 | 0 | 1 | 0 | 0 | 0 | 0 | 0 | 0 | 0 | 0 | 1 | 0 | 1 |
| ZCWPW1 | 1 | 0 | 1 | 0 | 2 | 0 | 0 | 0 | 0 | 0 | 0 | 0 | 0 | 1 | 0 | 1 |

| **ensembl gene name** | **MP_0009208_list** | **Mouse_gene_name** | **Human_gene_name** | **# MP Terms** | **# HPO Terms** | **TranscriptId** | **BioType** | **variants_impact_HIGH** | **variants_impact_LOW** | **variants_impact_MODERATE** | **variants_impact_MODIFIER** |
| --- | --- | --- | --- | --- | --- | --- | --- | --- | --- | --- | --- |
| ADAMTS3 | 0 | NA | ADAMTS3 | NA | 59 | ENSMMUT00000025259.3 | protein_coding | 0 | 25 | 16 | 2929 |
| AIRE | 0 | NA | AIRE | NA | 45 | ENSMMUT00000005921.3 | protein_coding | 0 | 33 | 17 | 517 |
| **ASPM** | **1** | **Aspm** | **ASPM** | **30** | **32** | **ENSMMUT00000000361.3** | **protein_coding** | **0** | **56** | **85** | **412** |
| BTBD8 | 0 | Btbd8 | NA | 7 | NA | ENSMMUT00000010026.3 | protein_coding | 0 | 22 | 34 | 559 |
| CAMKV | 1 | Camkv | NA | 6 | NA | ENSMMUT00000023149.3 | protein_coding | 0 | 8 | 0 | 109 |
| CCR8 | 1 | Ccr8 | NA | 5 | NA | ENSMMUT00000032322.3 | protein_coding | 0 | 7 | 5 | 86 |
| CD96 | 0 | NA | CD96 | NA | 80 | ENSMMUT00000000989.3 | protein_coding | 0 | 20 | 23 | 1138 |
| CEP19 | 0 | NA | CEP19 | NA | 39 | ENSMMUT00000020765.3 | protein_coding | 1 | 7 | 0 | 95 |
| CFAP44 | 0 | Cfap44 | NA | 17 | NA | ENSMMUT00000018871.3 | protein_coding | 0 | 36 | 21 | 863 |
| CPLANE1 | 0 | NA | CPLANE1 | NA | 92 | ENSMMUT00000064914.1 | protein_coding | 0 | 59 | 77 | 716 |
| CSPP1 | 0 | NA | CSPP1 | NA | 151 | ENSMMUT00000008790.3 | protein_coding | 1 | 19 | 15 | 698 |
| **CYP17A1** | **1** | **Cyp17a1** | **CYP17A1** | **29** | **68** | **ENSMMUT00000024752.3** | **protein_coding** | **0** | **11** | **2** | **90** |
| **CYP21A2** | **0** | **NA** | **CYP21A2** | **NA** | **12** | **ENSMMUT00000001089.3** | **protein_coding** | **2** | **32** | **21** | **446** |
| **DACT1** | **1** | **Dact1** | **DACT1** | **59** | **80** | **ENSMMUT00000001545.3** | **protein_coding** | **0** | **26** | **20** | **223** |
| DND1 | 1 | Dnd1 | NA | 24 | NA | ENSMMUT00000006346.3 | protein_coding | 0 | 7 | 2 | 93 |
| DPY19L2 | 0 | Dpy19l2 | NA | 16 | NA | ENSMMUT00000007133.3 | protein_coding | 0 | 36 | 37 | 1337 |
| EXO1 | 1 | Exo1 | NA | 28 | NA | ENSMMUT00000044472.2 | protein_coding | 0 | 33 | 28 | 478 |
| EYS | 0 | NA | EYS | NA | 14 | ENSMMUT00000063301.1 | protein_coding | 2 | 69 | 135 | 18395 |
| FAS | 1 | Fas | FAS | 190 | 149 | ENSMMUT00000055497.1 | protein_coding | 0 | 6 | 9 | 418 |
| **GATA3** | **0** | **NA** | **GATA3** | **NA** | **40** | **ENSMMUT00000009704.3** | **protein_coding** | **0** | **8** | **1** | **454** |
| GFM2 | 0 | NA | GFM2 | NA | 62 | ENSMMUT00000028107.3 | protein_coding | 0 | 10 | 12 | 381 |
| **HFE** | **0** | **NA** | **HFE** | **NA** | **79** | **ENSMMUT00000072973.1** | **protein_coding** | **0** | **7** | **4** | **178** |
| HFM1 | 1 | Hfm1 | HFM1 | 22 | 4 | ENSMMUT00000009536.3 | protein_coding | 0 | 23 | 32 | 795 |
| HMGA1 | 1 | Hmga1 | NA | 43 | NA | ENSMMUT00000027435.3 | protein_coding | 0 | 2 | 0 | 196 |
| HSPA4 | 0 | Hspa4 | NA | 17 | NA | ENSMMUT00000059541.1 | protein_coding | 0 | 19 | 6 | 449 |
| IL10 | 0 | NA | IL10 | NA | 79 | ENSMMUT00000033151.3 | protein_coding | 0 | 2 | 1 | 108 |
| INPP5B | 0 | Inpp5b | NA | 8 | NA | ENSMMUT00000069187.1 | protein_coding | 0 | 25 | 18 | 444 |
| KDM3B | 0 | NA | KDM3B | NA | 34 | ENSMMUT00000000668.3 | protein_coding | 0 | 27 | 7 | 411 |
| KIAA0586 | 0 | NA | KIAA0586 | NA | 127 | ENSMMUT00000001541.3 | protein_coding | 0 | 34 | 28 | 858 |
| MAB21L2 | 0 | NA | MAB21L2 | NA | 21 | ENSMMUT00000057185.1 | protein_coding | 0 | 5 | 0 | 155 |
| MAN2B2 | 1 | Man2b2 | NA | 9 | NA | ENSMMUT00000007452.3 | protein_coding | 1 | 31 | 27 | 383 |
| MANBA | 0 | Manba | NA | 17 | NA | ENSMMUT00000054639.1 | protein_coding | 0 | 17 | 17 | 1116 |
| MINPP1 | 0 | NA | MINPP1 | NA | 55 | ENSMMUT00000006730.3 | protein_coding | 0 | 14 | 10 | 460 |
| MST1R | 1 | Mst1r | NA | 21 | NA | ENSMMUT00000071891.1 | protein_coding | 0 | 27 | 17 | 179 |
| MYLK | 0 | NA | MYLK | NA | 66 | ENSMMUT00000046835.2 | protein_coding | 0 | 44 | 26 | 2178 |
| NODAL | 0 | NA | NODAL | NA | 143 | ENSMMUT00000032590.3 | protein_coding | 0 | 6 | 6 | 171 |
| NPHP4 | 0 | NA | NPHP4 | NA | 29 | ENSMMUT00000047628.2 | protein_coding | 0 | 86 | 48 | 2277 |
| NR2F2 | 1 | Nr2f2 | NR2F2 | 83 | 33 | ENSMMUT00000068442.1 | protein_coding | 0 | 6 | 0 | 115 |
| PCNT | 0 | NA | PCNT | NA | 102 | ENSMMUT00000018114.3 | protein_coding | 0 | 110 | 105 | 1747 |
| PGBD1 | 1 | Pgbd1 | NA | 15 | NA | ENSMMUT00000010102.3 | protein_coding | 0 | 18 | 15 | 157 |
| PIK3CA | 1 | Pik3ca | PIK3CA | 96 | 302 | ENSMMUT00000063241.1 | protein_coding | 0 | 21 | 0 | 312 |
| POU1F1 | 0 | NA | POU1F1 | NA | 86 | ENSMMUT00000070446.1 | protein_coding | 0 | 11 | 1 | 428 |
| PRPF3 | 0 | NA | PRPF3 | NA | 32 | ENSMMUT00000022895.3 | protein_coding | 0 | 12 | 1 | 138 |
| PSMB8 | 0 | NA | PSMB8 | NA | 77 | ENSMMUT00000032003.3 | protein_coding | 0 | 21 | 14 | 569 |
| PTPN23 | 0 | NA | PTPN23 | NA | 91 | ENSMMUT00000019713.3 | protein_coding | 0 | 46 | 24 | 368 |
| **PTPRC** | **0** | **NA** | **PTPRC** | **NA** | **13** | **ENSMMUT00000000014.3** | **protein_coding** | **0** | **36** | **69** | **1269** |
| RAF1 | 0 | NA | RAF1 | NA | 124 | ENSMMUT00000072482.1 | protein_coding | 0 | 11 | 2 | 338 |
| RF00108 | 0 | NA | SNORD116-1 | NA | 115 | ENSMMUT00000061111.1 | protein_coding | 0 | 0 | 0 | 236 |
| RNF148 | 0 | Rnf148 | NA | 7 | NA | ENSMMUT00000067367.1 | protein_coding | 0 | 2 | 1 | 135 |
| RP1L1 | 0 | NA | RP1L1 | NA | 39 | ENSMMUT00000071832.1 | protein_coding | 1 | 76 | 121 | 246 |
| SDCCAG8 | 0 | NA | SDCCAG8 | NA | 54 | ENSMMUT00000069525.1 | protein_coding | 1 | 18 | 17 | 2441 |
| SIN3A | 0 | NA | SIN3A | NA | 126 | ENSMMUT00000012974.3 | protein_coding | 0 | 25 | 8 | 585 |
| SKIDA1 | 1 | Skida1 | NA | 12 | NA | ENSMMUT00000033267.3 | protein_coding | 0 | 5 | 6 | 97 |
| SLC35D1 | 0 | NA | SLC35D1 | NA | 35 | ENSMMUT00000000931.2 | protein_coding | 0 | 6 | 1 | 449 |
| SLC9A3 | 0 | Slc9a3 | NA | 16 | NA | ENSMMUT00000005362.3 | protein_coding | 0 | 31 | 10 | 1266 |
| SNAI2 | 0 | Snai2 | NA | 41 | NA | ENSMMUT00000022753.3 | protein_coding | 0 | 7 | 0 | 277 |
| STAG1 | 1 | Stag1 | STAG1 | 47 | 43 | ENSMMUT00000071115.1 | protein_coding | 0 | 11 | 2 | 1308 |
| TACSTD2 | 0 | Tacstd2 | NA | 7 | NA | ENSMMUT00000032032.2 | protein_coding | 0 | 11 | 3 | 122 |
| TDRKH | 0 | Tdrkh | NA | 11 | NA | ENSMMUT00000073736.1 | protein_coding | 0 | 8 | 3 | 171 |
| TFAP2A | 0 | NA | TFAP2A | NA | 97 | ENSMMUT00000070579.1 | protein_coding | 0 | 7 | 0 | 485 |
| TLR2 | 0 | NA | TLR2 | NA | 8 | ENSMMUT00000022268.3 | protein_coding | 0 | 11 | 24 | 158 |
| TMED10 | 0 | Tmed10 | NA | 10 | NA | ENSMMUT00000068664.1 | protein_coding | 0 | 2 | 1 | 336 |
| TOGARAM1 | 0 | NA | TOGARAM1 | NA | 35 | ENSMMUT00000015191.3 | protein_coding | 0 | 28 | 25 | 502 |
| TOPAZ1 | 0 | Topaz1 | NA | 9 | NA | ENSMMUT00000060635.1 | protein_coding | 0 | 24 | 36 | 542 |
| UBE2D2 | 0 | Ube2d2a | NA | 1 | NA | ENSMMUT00000058323.1 | protein_coding | 0 | 2 | 0 | 135 |
| WDR48 | 0 | Wdr48 | NA | 14 | NA | ENSMMUT00000028337.3 | protein_coding | 0 | 8 | 0 | 396 |
| ZCWPW1 | 1 | Zcwpw1 | NA | 13 | NA | ENSMMUT00000042441.2 | protein_coding | 0 | 8 | 9 | 149 |

| **ensembl gene name** | **variants_effect_3_prime_UTR_variant** | **variants_effect_5_prime_UTR_premature_start_codon_gain_** | **variants_effect_5_prime_UTR_variant** | **variants_effect_downstream_gene_variant** | **variants_effect_initiator_codon_variant** | **variants_effect_intron_variant** |
| --- | --- | --- | --- | --- | --- | --- |
| ADAMTS3 | 36 | 1 | 2 | 69 | 0 | 2762 |
| AIRE | 7 | 4 | 19 | 60 | 0 | 321 |
| **ASPM** | **0** | **0** | **0** | **32** | **0** | **362** |
| BTBD8 | 2 | 1 | 8 | 30 | 0 | 495 |
| CAMKV | 0 | 0 | 0 | 46 | 0 | 25 |
| CCR8 | 0 | 3 | 5 | 26 | 0 | 12 |
| CD96 | 0 | 0 | 0 | 116 | 0 | 933 |
| CEP19 | 0 | 3 | 10 | 21 | 0 | 27 |
| CFAP44 | 0 | 0 | 0 | 99 | 0 | 738 |
| CPLANE1 | 0 | 2 | 5 | 12 | 0 | 660 |
| CSPP1 | 0 | 0 | 0 | 74 | 0 | 587 |
| **CYP17A1** | **0** | **0** | **0** | **15** | **0** | **70** |
| **CYP21A2** | **0** | **0** | **0** | **243** | **0** | **53** |
| **DACT1** | **24** | **1** | **4** | **32** | **0** | **101** |
| DND1 | 9 | 0 | 1 | 27 | 0 | 5 |
| DPY19L2 | 0 | 3 | 17 | 157 | 0 | 970 |
| EXO1 | 12 | 0 | 12 | 16 | 0 | 374 |
| EYS | 0 | 0 | 0 | 57 | 0 | 18278 |
| FAS | 0 | 0 | 0 | 30 | 0 | 320 |
| **GATA3** | **0** | **0** | **0** | **49** | **0** | **310** |
| GFM2 | 0 | 2 | 8 | 44 | 0 | 293 |
| **HFE** | **29** | **2** | **6** | **77** | **0** | **57** |
| HFM1 | 0 | 1 | 2 | 23 | 0 | 738 |
| HMGA1 | 8 | 0 | 0 | 63 | 0 | 82 |
| HSPA4 | 37 | 0 | 0 | 53 | 0 | 322 |
| IL10 | 2 | 0 | 0 | 23 | 0 | 51 |
| INPP5B | 0 | 0 | 0 | 53 | 0 | 312 |
| KDM3B | 12 | 0 | 1 | 47 | 0 | 298 |
| KIAA0586 | 0 | 0 | 2 | 34 | 0 | 787 |
| MAB21L2 | 0 | 0 | 0 | 83 | 0 | 0 |
| MAN2B2 | 22 | 0 | 2 | 4 | 0 | 346 |
| MANBA | 0 | 0 | 0 | 57 | 0 | 1056 |
| MINPP1 | 0 | 0 | 4 | 80 | 0 | 287 |
| MST1R | 19 | 1 | 5 | 34 | 0 | 86 |
| MYLK | 25 | 0 | 0 | 32 | 0 | 2126 |
| NODAL | 0 | 0 | 0 | 61 | 0 | 67 |
| NPHP4 | 23 | 5 | 25 | 192 | 0 | 1909 |
| NR2F2 | 0 | 0 | 0 | 38 | 0 | 43 |
| PCNT | 0 | 0 | 0 | 13 | 0 | 1702 |
| PGBD1 | 3 | 10 | 36 | 30 | 0 | 63 |
| PIK3CA | 0 | 0 | 1 | 42 | 0 | 233 |
| POU1F1 | 0 | 1 | 9 | 77 | 0 | 290 |
| PRPF3 | 0 | 0 | 0 | 15 | 0 | 107 |
| PSMB8 | 90 | 8 | 55 | 171 | 0 | 89 |
| PTPN23 | 1 | 0 | 1 | 85 | 0 | 255 |
| **PTPRC** | **0** | **0** | **0** | **72** | **0** | **1163** |
| RAF1 | 0 | 0 | 0 | 53 | 0 | 269 |
| RF00108 | 0 | 0 | 0 | 185 | 0 | 0 |
| RNF148 | 0 | 0 | 0 | 78 | 0 | 2 |
| RP1L1 | 0 | 0 | 0 | 89 | 0 | 50 |
| SDCCAG8 | 61 | 0 | 0 | 86 | 0 | 2224 |
| SIN3A | 6 | 0 | 2 | 66 | 0 | 445 |
| SKIDA1 | 0 | 0 | 0 | 52 | 0 | 0 |
| SLC35D1 | 0 | 0 | 0 | 49 | 0 | 353 |
| SLC9A3 | 0 | 0 | 0 | 110 | 0 | 1095 |
| SNAI2 | 56 | 0 | 2 | 44 | 0 | 47 |
| STAG1 | 0 | 0 | 0 | 52 | 0 | 1257 |
| TACSTD2 | 15 | 3 | 6 | 56 | 0 | 2 |
| TDRKH | 0 | 0 | 0 | 43 | 0 | 105 |
| TFAP2A | 18 | 0 | 0 | 71 | 0 | 273 |
| TLR2 | 2 | 2 | 5 | 39 | 0 | 40 |
| TMED10 | 29 | 0 | 0 | 42 | 0 | 242 |
| TOGARAM1 | 11 | 0 | 0 | 25 | 0 | 402 |
| TOPAZ1 | 0 | 0 | 0 | 14 | 0 | 491 |
| UBE2D2 | 0 | 0 | 0 | 28 | 0 | 97 |
| WDR48 | 21 | 0 | 2 | 60 | 0 | 296 |
| ZCWPW1 | 25 | 0 | 1 | 27 | 0 | 88 |

| **ensembl gene name** | **variants_effect_missense_variant** | **variants_effect_non_coding_transcript_exon_variant** | **variants_effect_splice_acceptor_variant** | **variants_effect_splice_donor_variant** | **variants_effect_splice_region_variant** | **variants_effect_start_lost** | **variants_effect_stop_gained** |
| --- | --- | --- | --- | --- | --- | --- | --- |
| ADAMTS3 | 16 | 0 | 0 | 0 | 3 | 0 | 0 |
| AIRE | 17 | 0 | 0 | 0 | 7 | 0 | 0 |
| **ASPM** | **85** | **0** | **0** | **0** | **7** | **0** | **0** |
| BTBD8 | 34 | 0 | 0 | 0 | 1 | 0 | 0 |
| CAMKV | 0 | 0 | 0 | 0 | 3 | 0 | 0 |
| CCR8 | 5 | 0 | 0 | 0 | 0 | 0 | 0 |
| CD96 | 23 | 0 | 0 | 0 | 2 | 0 | 0 |
| CEP19 | 0 | 0 | 0 | 0 | 1 | 1 | 0 |
| CFAP44 | 21 | 0 | 0 | 0 | 4 | 0 | 0 |
| CPLANE1 | 77 | 0 | 0 | 0 | 8 | 0 | 0 |
| CSPP1 | 15 | 0 | 0 | 1 | 2 | 0 | 0 |
| **CYP17A1** | **2** | **0** | **0** | **0** | **3** | **0** | **0** |
| **CYP21A2** | **21** | **0** | **0** | **0** | **7** | **0** | **2** |
| **DACT1** | **20** | **0** | **0** | **0** | **0** | **0** | **0** |
| DND1 | 2 | 0 | 0 | 0 | 0 | 0 | 0 |
| DPY19L2 | 37 | 0 | 0 | 0 | 9 | 0 | 0 |
| EXO1 | 28 | 0 | 0 | 0 | 0 | 0 | 0 |
| EYS | 135 | 0 | 0 | 1 | 12 | 0 | 1 |
| FAS | 9 | 0 | 0 | 0 | 1 | 0 | 0 |
| **GATA3** | **1** | **0** | **0** | **0** | **0** | **0** | **0** |
| GFM2 | 12 | 0 | 0 | 0 | 4 | 0 | 0 |
| **HFE** | **4** | **0** | **0** | **0** | **2** | **0** | **0** |
| HFM1 | 32 | 0 | 0 | 0 | 7 | 0 | 0 |
| HMGA1 | 0 | 0 | 0 | 0 | 2 | 0 | 0 |
| HSPA4 | 6 | 0 | 0 | 0 | 3 | 0 | 0 |
| IL10 | 1 | 0 | 0 | 0 | 1 | 0 | 0 |
| INPP5B | 18 | 0 | 0 | 0 | 6 | 0 | 0 |
| KDM3B | 7 | 0 | 0 | 0 | 2 | 0 | 0 |
| KIAA0586 | 28 | 0 | 0 | 0 | 9 | 0 | 0 |
| MAB21L2 | 0 | 0 | 0 | 0 | 0 | 0 | 0 |
| MAN2B2 | 27 | 0 | 1 | 0 | 4 | 0 | 0 |
| MANBA | 17 | 0 | 0 | 0 | 6 | 0 | 0 |
| MINPP1 | 10 | 0 | 0 | 0 | 2 | 0 | 0 |
| MST1R | 17 | 0 | 0 | 0 | 1 | 0 | 0 |
| MYLK | 26 | 0 | 0 | 0 | 7 | 0 | 0 |
| NODAL | 6 | 0 | 0 | 0 | 1 | 0 | 0 |
| NPHP4 | 48 | 0 | 0 | 0 | 16 | 0 | 0 |
| NR2F2 | 0 | 0 | 0 | 0 | 0 | 0 | 0 |
| PCNT | 105 | 0 | 0 | 0 | 12 | 0 | 0 |
| PGBD1 | 15 | 0 | 0 | 0 | 2 | 0 | 0 |
| PIK3CA | 0 | 0 | 0 | 0 | 5 | 0 | 0 |
| POU1F1 | 1 | 0 | 0 | 0 | 1 | 0 | 0 |
| PRPF3 | 1 | 0 | 0 | 0 | 4 | 0 | 0 |
| PSMB8 | 14 | 0 | 0 | 0 | 1 | 0 | 0 |
| PTPN23 | 24 | 0 | 0 | 0 | 7 | 0 | 0 |
| **PTPRC** | **69** | **0** | **0** | **0** | **6** | **0** | **0** |
| RAF1 | 2 | 0 | 0 | 0 | 2 | 0 | 0 |
| RF00108 | 0 | 4 | 0 | 0 | 0 | 0 | 0 |
| RNF148 | 1 | 0 | 0 | 0 | 0 | 0 | 0 |
| RP1L1 | 121 | 0 | 0 | 0 | 0 | 0 | 1 |
| SDCCAG8 | 17 | 0 | 0 | 0 | 2 | 0 | 1 |
| SIN3A | 8 | 0 | 0 | 0 | 3 | 0 | 0 |
| SKIDA1 | 6 | 0 | 0 | 0 | 0 | 0 | 0 |
| SLC35D1 | 1 | 0 | 0 | 0 | 1 | 0 | 0 |
| SLC9A3 | 10 | 0 | 0 | 0 | 6 | 0 | 0 |
| SNAI2 | 0 | 0 | 0 | 0 | 1 | 0 | 0 |
| STAG1 | 2 | 0 | 0 | 0 | 1 | 0 | 0 |
| TACSTD2 | 3 | 0 | 0 | 0 | 0 | 0 | 0 |
| TDRKH | 3 | 0 | 0 | 0 | 3 | 0 | 0 |
| TFAP2A | 0 | 0 | 0 | 0 | 3 | 0 | 0 |
| TLR2 | 24 | 0 | 0 | 0 | 1 | 0 | 0 |
| TMED10 | 1 | 0 | 0 | 0 | 1 | 0 | 0 |
| TOGARAM1 | 25 | 0 | 0 | 0 | 0 | 0 | 0 |
| TOPAZ1 | 36 | 0 | 0 | 0 | 1 | 0 | 0 |
| UBE2D2 | 0 | 0 | 0 | 0 | 1 | 0 | 0 |
| WDR48 | 0 | 0 | 0 | 0 | 2 | 0 | 0 |
| ZCWPW1 | 9 | 0 | 0 | 0 | 0 | 0 | 0 |
